# Comparative Genome Mining of *Jiangella* sp. GA3 from Turtle-Ingested Plastic Reveals Conserved Biosynthetic Gene Clusters

**DOI:** 10.3390/md24090319

**Published:** 2026-09-10

**Authors:** Ruggero Baviera, Fanny Claire Capri, Paola Galluzzo, Rosa Alduina

**Affiliations:** 1 Dipartimento Scienze e Tecnologia Biologiche, Chimiche e Farmaceutiche, Viale delle Scienze, Università Di Palermo, 90133 Palermo, Italy; ruggero.baviera@community.unipa.it; 2 National Biodiversity Future Center (NBFC), Piazza Marina, 61, 90133 Palermo, Italy; 3 Istituto Zooprofilattico Sperimentale della Sicilia “Mirri”, Via G. Marinuzzi 3, 90129 Palermo, Italy; paola.galluzzo@izssicilia.it

**Keywords:** *Jiangella*, rare actinobacteria, genome mining, biosynthetic gene clusters, BiG-SCAPE, BiNI, RiPP, lasso peptide, plastisphere, *Caretta caretta*

## Abstract

Rare actinobacteria are promising sources of specialized metabolites, yet genome mining still prioritizes genomes encoding many biosynthetic gene clusters (BGCs) rather than clusters that are distant from characterized chemistry. Here, we describe the isolation of a novel *Jiangella* strain reported from a marine, animal-associated plastisphere and place its biosynthetic repertoire in the context of the whole genus, providing a genome-based prioritization of candidate biosynthetic targets. Digital DNA-DNA hybridization (57.8%) and OrthoANIu (94.65%) values against its closest relative, *J. alba* DSM 45237ᵀ, support its assignment as a candidate novel species, although formal species description will require additional phenotypic and chemotaxonomic characterization. antiSMASH predicted twelve BGCs, within the range observed across the genus (7–13), of which only one matched a characterized pathway, and the Biosynthetic Novelty Index of the genome was 865.3, the fourth highest among the twelve genomes analyzed. Across these genomes, 124 BGCs grouped into 70 gene cluster families, 74% of them strain-specific. Two families without any characterized representative, a lasso-peptide family and a RiPP recognition element-containing family, were instead conserved across a subclade, down to nearly identical precursor peptides. No antimicrobial activity was detected under the conditions tested. Genus-wide comparison therefore identifies conserved yet uncharacterized families as rational priorities that cluster counts alone would miss.

## 1. Introduction

Rare actinobacteria genera are recovered far less frequently than *Streptomyces* under standard cultivation conditions and, correspondingly, are less studied and are of interest for natural product discovery not for the quantity of specialized metabolism they encode, but because their evolutionary distance from well-studied taxa makes their chemistry more likely to differ from what is already known [1]. In bacteria, the genes responsible for the biosynthesis of specialized metabolites are organized in biosynthetic gene clusters (BGCs), and clusters encoding related pathways in different strains can be grouped into gene cluster families (GCFs) [2]. Genome mining now predicts more BGCs than can be experimentally investigated, shifting the bottleneck from detection to selection [3]. Genomes are commonly ranked by the number of predicted clusters, a criterion that favors biosynthetically prolific taxa and is insensitive to how far those clusters lie from characterized chemistry. The Biosynthetic Novelty Index (BiNI) was introduced to address this limitation, weighting each predicted cluster according to its distance from known gene cluster families rather than counting clusters, thereby providing a more informative measure of biosynthetic novelty [4].

Biosynthetic novelty alone, however, provides only part of the picture. Without an evolutionary framework, it is difficult to establish whether a predicted cluster represents a conserved feature of a lineage, a recent strain-specific acquisition, or, particularly in draft genomes, a prediction artifact. Comparative analyses across multiple genomes of the same genus resolve this ambiguity, as a gene cluster family recovered independently from several assemblies is unlikely to be an artifact, and its distribution can be interpreted against the phylogeny of the group. Evolutionary information of this kind is one of the criteria proposed for prioritizing BGCs [5], and genus-wide comparisons have been carried out in *Salinispora* [6], *Amycolatopsis* [7] and *Streptomyces* [8]. These studies revealed a recurring organization, in which a small set of families conserved across the genus coexists with a much larger accessory fraction: the conserved core is largely inherited vertically, whereas horizontal transfer contributes substantially to the accessory repertoire, in proportions that differ markedly between genera. Conservation, on its own, says nothing about whether a family has been studied: a family may recur throughout a genus and still have no experimentally characterized representative. This combination is informative, because recurrence across independently sequenced species indicates that the cluster is real and has been maintained by the lineage, whereas the absence of a characterized representative indicates that its product is unknown. Families with both features are therefore rational starting points for experimental work.

Novel actinobacterial taxa encoding uncharacterized biosynthetic pathways continue to be described from extreme and poorly sampled habitats [9]. One such underexplored niche is marine plastic debris, a durable artificial substrate that is rapidly colonized by complex microbial biofilms, collectively referred to as the plastisphere [10]. While culture-independent surveys have revealed considerable taxonomic diversity within these communities [10,11], culture-dependent work has concentrated on pathogens and antibiotic-resistant bacteria [12] and on polymer-degrading strains [13]. Their capacity to produce specialized metabolites from this niche has, by contrast, received little attention. Plastic debris recovered from the digestive tract of marine vertebrates represents an even less explored niche. Loggerhead sea turtles (*Caretta caretta*), which frequently ingest plastic debris and are widely recognized as sentinels of marine litter in the Mediterranean Sea [14], retain ingested fragments in the digestive tract for prolonged periods [15], and the biofilms recovered from them have therefore been exposed to the host gut environment-a combination of substrate and environment that has not been examined as a source of biosynthetically novel actinobacteria.

Following this lead, we isolated a strain of *Jiangella* from plastic debris recovered from the feces of a Mediterranean loggerhead sea turtle. The genus *Jiangella* comprises a rare actinobacterial lineage first described in 2005 [16], currently including eleven validly published species recovered from a wide range of different environments, including arid and desert soils, caves and mangrove sediments, as well as from plant tissues [16,17,18,19]. The jiangrines and jiangolide, isolated from *Jiangella gansuensis,* are the first specialized metabolites reported from the entire order Jiangellales, and most of them showed anti-inflammatory activity [20,21]. The genus therefore remains almost entirely unexplored from a biosynthetic perspective, and its specialized metabolism has never been examined within a comparative evolutionary framework.

In the present study, we characterized strain GA3 phenotypically and by whole-genome sequencing and placed its biosynthetic repertoire in the context of all publicly available *Jiangella* genomes meeting our quality criteria. Our aims were to establish the taxonomic position of the strain, to annotate its BGCs with complementary detection tools and quantify their distance from characterized cluster families across the genus, to describe how biosynthetic diversity is organized within *Jiangella*, and to identify cluster families that are conserved across the genus yet lack any characterized representative. The study represents therefore a genome-guided prioritization framework for subsequent chemical and functional characterization.

## 2. Results

### 2.1. Morphological and Phenotypic Characterization

**Figure 1:**
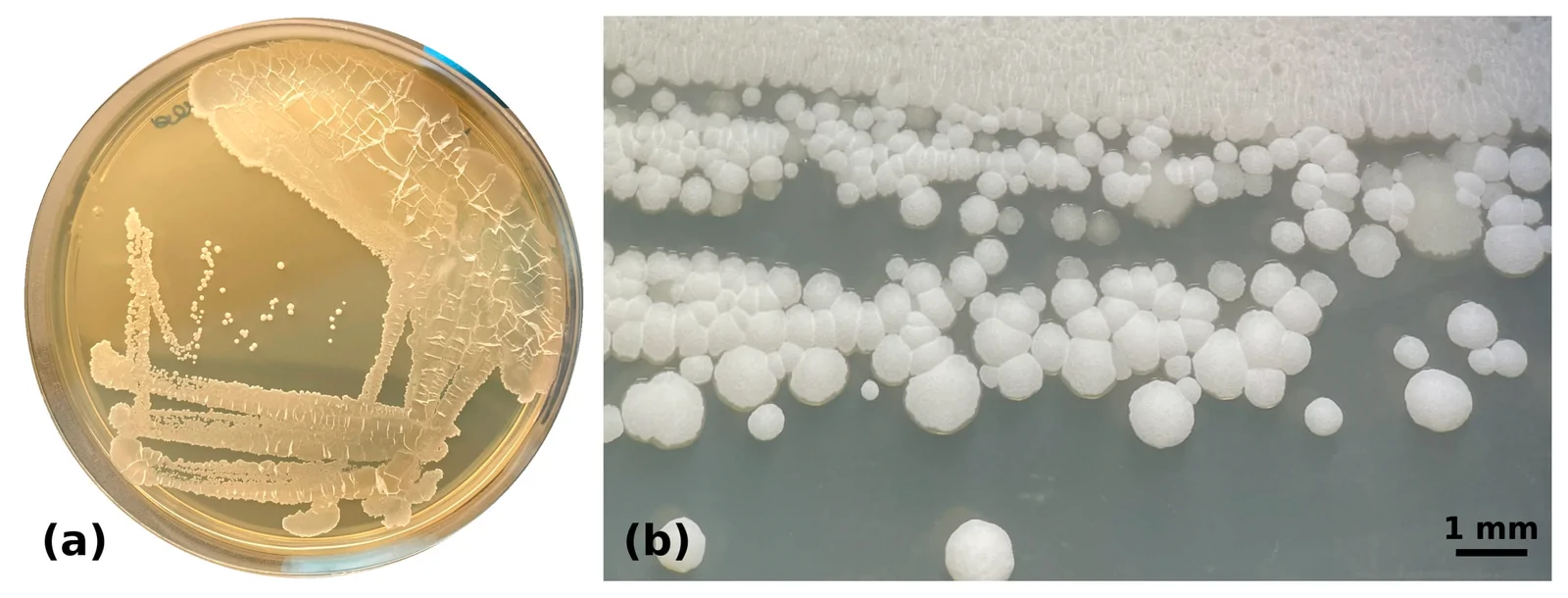
Colony morphology of *Jiangella* sp. GA3. (**a**) Macroscopic growth on ISP2 agar after 21 days at 30 °C; (**b**) magnification of single colonies after 10 days on tryptic soy agar, showing the thin white aerial mycelium. Scale bar in (**b**), 1 mm.

Strain GA3 was recovered as part of a broader collection of isolates from the plastisphere of *Caretta caretta*; the present study focuses on this isolate. 16S rRNA gene Sanger sequencing and further BLAST analysis assigned strain GA3 to the genus *Jiangella*. Strain GA3 showed morphological characteristics commonly present in the *Jiangella* genus. Colonies were cream to off-white (Figure 1a,b). A white and velvety colony phenotype was associated with the development of a thin aerial mycelium; margins were generally regular, although in some cases an irregular expansion was observed. Growth on ISP2 was abundant at 30 °C (+++), moderate at 37 °C (++), limited at 20 and 25 °C (+) and absent at 50 °C (−). No diffusible pigment was observed under any condition tested. No inhibition zone was observed in the agar plug diffusion assay, and no zone diameters were therefore recorded against *Kocuria rhizophila*, *Staphylococcus aureus* or *Escherichia coli*, on any of the four media tested.

### 2.2. Genome Analysis

**Figure 2:**
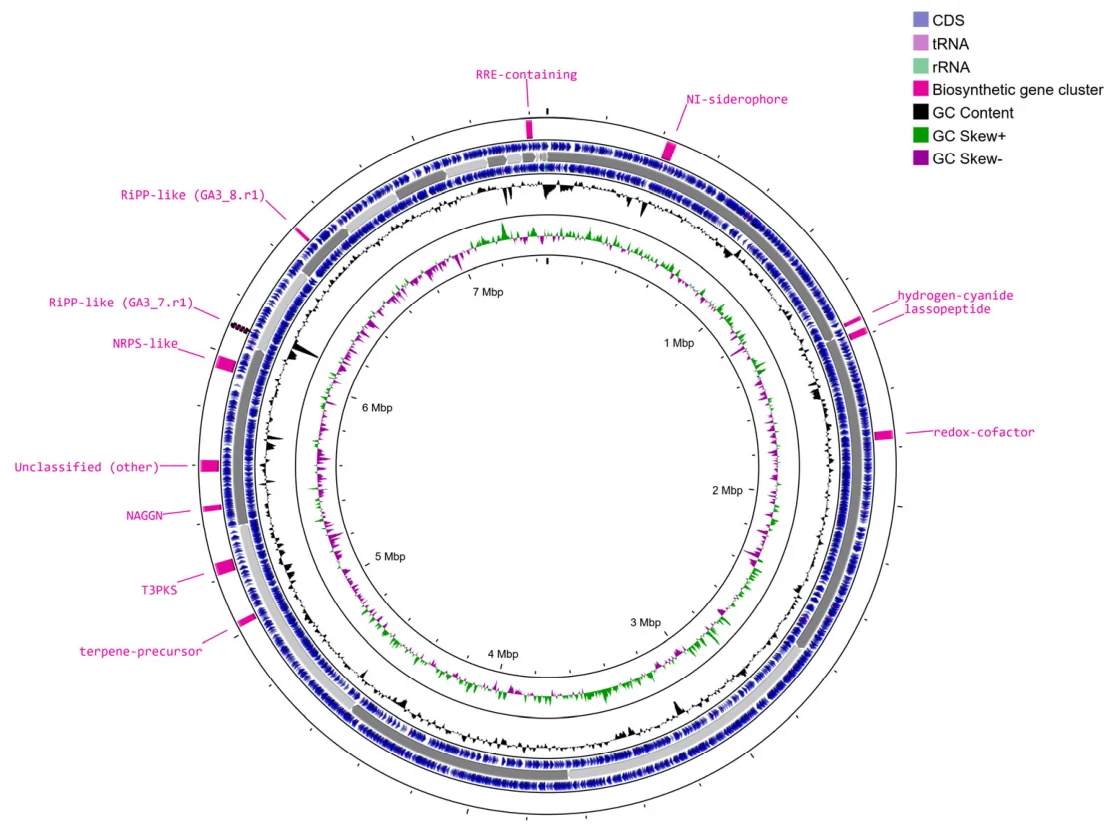
Circular map of the *Jiangella* sp. GA3 draft genome. From the outermost ring inwards: predicted biosynthetic gene clusters (magenta), labeled with their antiSMASH product class; protein-coding genes on the forward strand; the sequence backbone, in which alternating shades of grey mark the boundaries of the 25 annotated contigs (two contigs shorter than 1 kb were excluded before annotation); protein-coding genes on the reverse strand; GC content plotted as deviation from the genome mean of 72.81% (black); and GC skew, calculated as (G−C)/(G+C), with positive values in green and negative values in purple. Transfer RNA and ribosomal RNA genes are included in the coding rings. The position scale is given in Mb. Contig order in a draft assembly is arbitrary, and inter-contig distances are not biologically meaningful. Product classes are abbreviated as follows: NI-siderophore, non-ribosomal peptide synthetase (NRPS)-independent siderophore; T3PKS, type III polyketide synthase; NAGGN, *N*-acetylglutaminylglutamine amide; NRPS-like, non-ribosomal peptide synthetase-like; RiPP-like, ribosomally synthesized and post-translationally modified peptide-like; RRE-containing, RiPP recognition element-containing.

The draft genome of strain GA3 comprised 27 contigs totaling 7,462,732 bp, with the largest contig of 1,369,243 bp, an N50 of 886,138 bp (L50 = 4), and a G+C content of 72.81 mol%. Quality-filtered reads corresponded to an average genome coverage of 287×. Genome completeness and contamination estimated with CheckM were 100.00% and 0.59%, respectively. Annotation with PGAP, performed on the 25 contigs longer than 1 kb (7,461,120 bp), predicted 6815 genes, 46 tRNAs, and 3 rRNAs (Table 1, Figure 2).

Of the 6763 predicted proteins, 968 (14.3%) are annotated as hypothetical. The remainder indicate the metabolic capabilities expected of an aerobic chemoheterotroph, with a complete respiratory chain, a glyoxylate shunt and a complete pathway for nitrogen assimilation from nitrate to glutamate, comprising a respiratory nitrate reductase, a nitrite reductase, ammonium transporters, glutamine synthetase, glutamate synthase and glutamate dehydrogenases. A broad repertoire of hydrolytic enzymes is present, including glycoside hydrolases, glycosyltransferases, peptidases and proteases. Transport and regulation account for a large share of the coding capacity, with 706 ABC transporter components and 472 transcriptional regulators, alongside MFS transporters, sigma factors and two-component systems. Genes supporting redox and osmotic homeostasis include catalase, superoxide dismutase, thioredoxins and peroxiredoxins, the mycothiol biosynthetic pathway, trehalose biosynthesis and glycine betaine uptake. The genome carries seventeen transposase genes, from six insertion sequence families, eight of which are annotated as pseudogenes, alongside a limited complement of integrases, phage-related and conjugative transfer genes. Full counts are given in Table S1.

### 2.3. Phylogenomic Analysis and Species Delimitation

A nearly full-length 16S rRNA gene (~1517 bp) recovered from the assembly was most similar to that of *Jiangella alba* (99.80% identity), followed by other members of the genus *Jiangella* (Table S2). All best hits belonged to *Jiangella*, placing strain GA3 within this genus.

**Figure 3:**
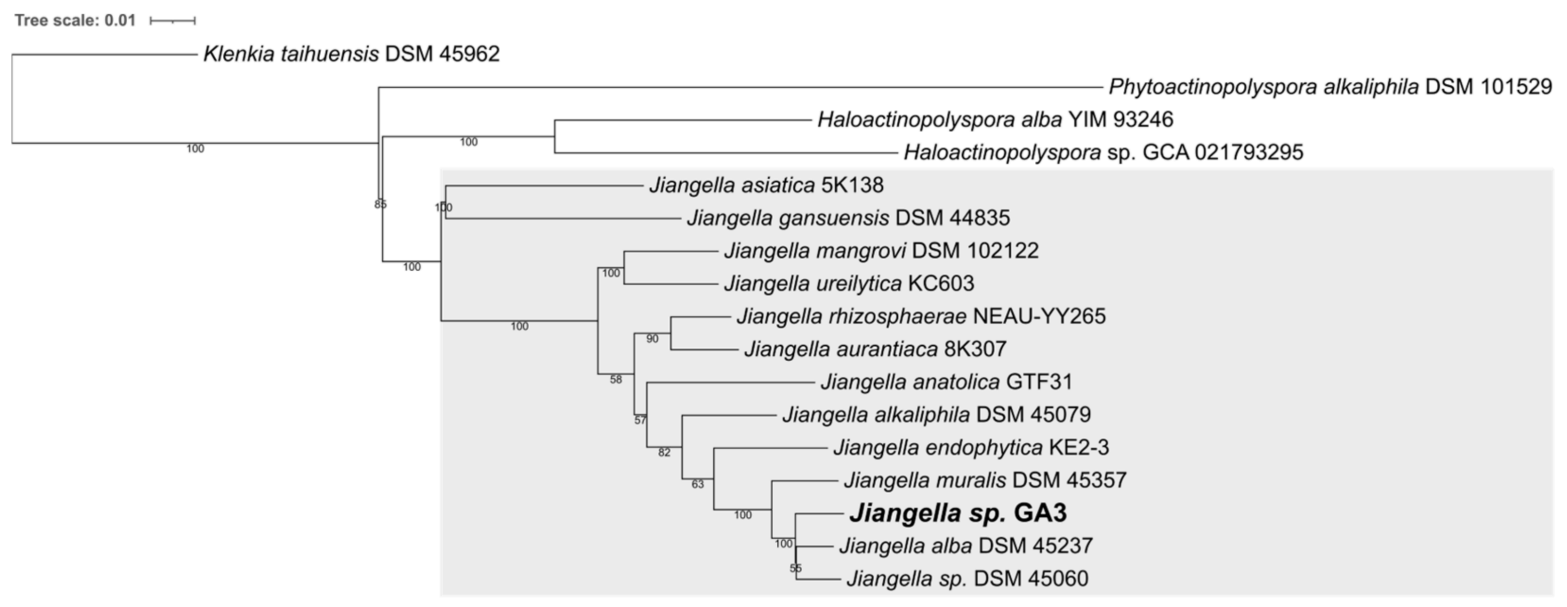
Maximum-likelihood phylogenomic tree of *Jiangella* sp. GA3 inferred from concatenated core genes with autoMLST2 (IQ-TREE). The tree was reconstructed using the full autoMLST reference set (52 genomes) and pruned to the 17 taxa shown; it is rooted on *Klenkia taihuensis*. The genus *Jiangella* is shaded in grey and strain GA3 is shown in bold. All *Jiangella* strains shown are the type strains of validly published species, with the exception of *Jiangella* sp. DSM 45060 and our strain GA3. Numbers at nodes indicate branch support (%); the scale bar represents 0.01 substitutions per site.

In the core-gene phylogeny (Figure 3), all validly published *Jiangella* type strains formed a strongly supported clade, with *Haloactinopolyspora* spp. as its sister group. Within this clade, strain GA3 was recovered in a robust sister relationship with the clade formed by the type strain of *J. alba* and *Jiangella* sp. DSM 45060 (bootstrap support 100%); this group joined *J. muralis* with equally strong support (100%), whereas the successive node including *J. endophytica* received moderate support (63%).

Classification with GTDB-Tk placed strain GA3 in the genus *Jiangella* without assigning it to any described species. dDDH values (formula d4) between strain GA3 and the type strains of all validly published *Jiangella* species were well below the 70% species threshold (Table 2). The highest value was obtained against *J. alba* DSM 45237ᵀ (57.8%), followed by *J. muralis* DSM 45357ᵀ (51.0%); all remaining type strains gave values ≤ 37.7%. Type-based species clustering in TYGS assigned GA3 to a distinct species cluster, not shared with any described *Jiangella* species.

Consistent with the dDDH results, the OrthoANIu value between GA3 and its closest relative, *J. alba* DSM 45237ᵀ, was 94.65%, followed by *J. muralis* DSM 45357ᵀ (93.23%) and *J. endophytica* KE2-3ᵀ (89.35%). Genome relatedness indices support GA3 as a candidate novel species within the genus *Jiangella*, although formal species description will require additional phenotypic and chemotaxonomic characterization.

### 2.4. Biosynthetic Potential

Genome mining with antiSMASH, performed on the same 25 contigs, predicted twelve BGCs in the genome of strain GA3, ranging from 10.8 to 42.5 kb and distributed over seven contigs, none truncated by a contig boundary (Table 3, Figure 2). One cluster showed high similarity to a characterized pathway, two showed low similarity, and the remaining nine had no close match in the MIBiG database. The BiNI of the genome was 865.3, with nine of the twelve clusters exceeding the novelty threshold (d > 900) [4]. DeepBGC independently predicted 100 candidate regions, 61 without an assigned product class and predicted antibacterial activity for 71 (Table S3).

The only cluster with a closely characterized counterpart was the type III polyketide synthase (T3PKS) cluster of region GA3_5.r2 (41.1 kb; category I), three of whose proteins shared 59–65% identity (KnownClusterBlast; Table S4) with SGR_470, SGR_471 and SGR_472 of the phenolic lipid biosynthetic cluster of *Streptomyces griseus* (MIBiG BGC0000282). Two further clusters retained only weak similarity to characterized pathways: a non-ribosomal peptide synthetase (NRPS)-independent siderophore (NI-siderophore) cluster (GA3_1.r1; 33.3 kb) related to schizokinen, and a hydrogen-cyanide cluster (GA3_1.r2; 12.8 kb) related to aborycin (both category I). In the former, the matching proteins are accessory biosynthetic enzymes of the siderophore pathway; in the latter, none of the three matching proteins is a biosynthetic gene (Table S4).

Five of the twelve clusters were assigned to RiPP or RiPP-associated classes. The lasso-peptide cluster of region GA3_2.r1 (22.5 kb; category II) encoded a complete set of predicted maturation enzymes-a lasso cyclase, a B2 leader peptidase and a PqqD-family RiPP recognition element-together with two distinct precursor peptides of 47 and 44 residues sharing a conserved leader motif (Table S5). RODEO identified both as the highest-scoring lasso precursors of the cluster (heuristic scores 10 and 9, respectively) and predicted nine-residue macrolactams with core masses of 2859 and 2718 Da, although its binary classifier scored both as negative. The redox-cofactor cluster of region GA3_2.r2 (29.5 kb; category III) corresponded to a complete predicted mycofactocin locus (*mftA–mftF*) [22], and was independently recovered by BAGEL4. Region GA3_14.r1 (20.3 kb; category II) was organized around a PqqD-family RiPP recognition element with a DUF6355-family protein as putative precursor, flanked by two methyltransferases, three glycosyltransferases, and an ABC transporter.

Of the two antiSMASH “RiPP-like” regions, GA3_8.r1 (10.8 kb) encoded a putative family-1 encapsulin shell protein together with a DyP-type peroxidase and a SigE-family sigma factor, and was therefore assigned to category III, while region GA3_7.r1 (11.5 kb) contained no identifiable precursor peptide and was assigned to category IV. A genome-wide BAGEL4 analysis recovered the mycofactocin and encapsulin loci but not the lasso-peptide or RiPP recognition element-containing clusters and returned no further RiPP locus with a recognizable precursor peptide.

The remaining four clusters had no close match to characterized pathways: a terpene-precursor cluster (GA3_5.r1; 21.1 kb, category I), an N-acetylglutaminylglutamine amide (NAGGN) cluster (GA3_6.r1; 18.2 kb, category III), an NRPS-like cluster (GA3_6.r3; 42.5 kb, category I) and an unclassified “other” region (GA3_6.r2; 40.5 kb, category IV).

### 2.5. Comparative Analysis of Biosynthetic Gene Clusters Across the Genus Jiangella

The number of BGCs per genome ranged from 7 to 13 (median 11); strain GA3, with 12, was exceeded only by *J. alba* (13). BiNI, calculated for all twelve genomes under identical conditions (Table S6), ranged from 335.0 in *J. anatolica* to 1217.4 in *J. mangrovi*, with a median of 787.6, and strain GA3 ranked fourth. Twenty-four of the 112 regions predicted in the reference genomes were reported as fragmented by BiG-FAM, concentrated in the least contiguous assemblies (Table S6). Their mean distance was lower than that of complete regions (898.5 against 959.8), so the index is underestimated in those genomes.

**Figure 4:**
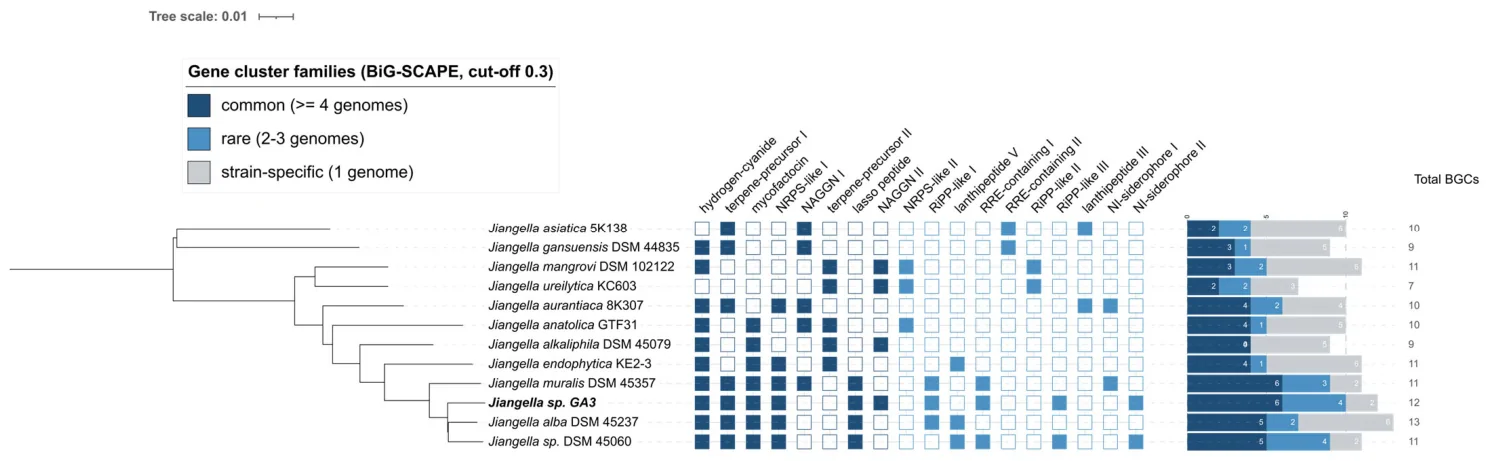
Distribution of biosynthetic gene cluster families across the genus *Jiangella*. The tree is the concatenated core-gene maximum-likelihood phylogeny of Figure 3, pruned to the twelve genomes included in the comparative analysis; *J. rhizosphaerae* is not represented because its assembly did not pass the contig-number filter applied in the Methods. Strain GA3 is shown in bold. The matrix shows the eighteen gene cluster families delineated with BiG-SCAPE v2.0.3 at a distance cut-off of 0.3 that are shared by two or more genomes; filled square indicates that the genome contains at least one cluster of that family. Families are labeled by their antiSMASH product class, and roman numerals distinguish different families assigned to the same class. Dark blue, families present in four or more genomes (common); light blue, families present in two or three genomes (rare). The fifty-two strain-specific families, each restricted to a single genome, are not shown individually in the matrix. The stacked bars on the right give the number of biosynthetic gene clusters (BGCs) predicted in each genome, divided into the same three categories (grey, strain-specific), with the total for each genome indicated at the far right.

The 124 BGC regions predicted across the twelve genomes were grouped into 70 GCFs at a distance cut-off of 0.3 (8 common, 10 rare, 52 strain-specific; Table S7), and 47 families at 0.5 (10 common, 6 rare, 31 strain-specific; Table S7). Several antiSMASH product classes were represented by more than one family shared by two or more genomes (Figure 4, Table S7). Of the twelve BGCs of strain GA3, ten fell into families shared with at least one other genome (six common and four rare at a cut-off of 0.3) and two formed strain-specific families. Across the dataset, the number of strain-specific families per genome ranged from two to six; strain GA3, *J. muralis*, and *Jiangella* sp. DSM 45060 each carried two.

**Figure 5:**
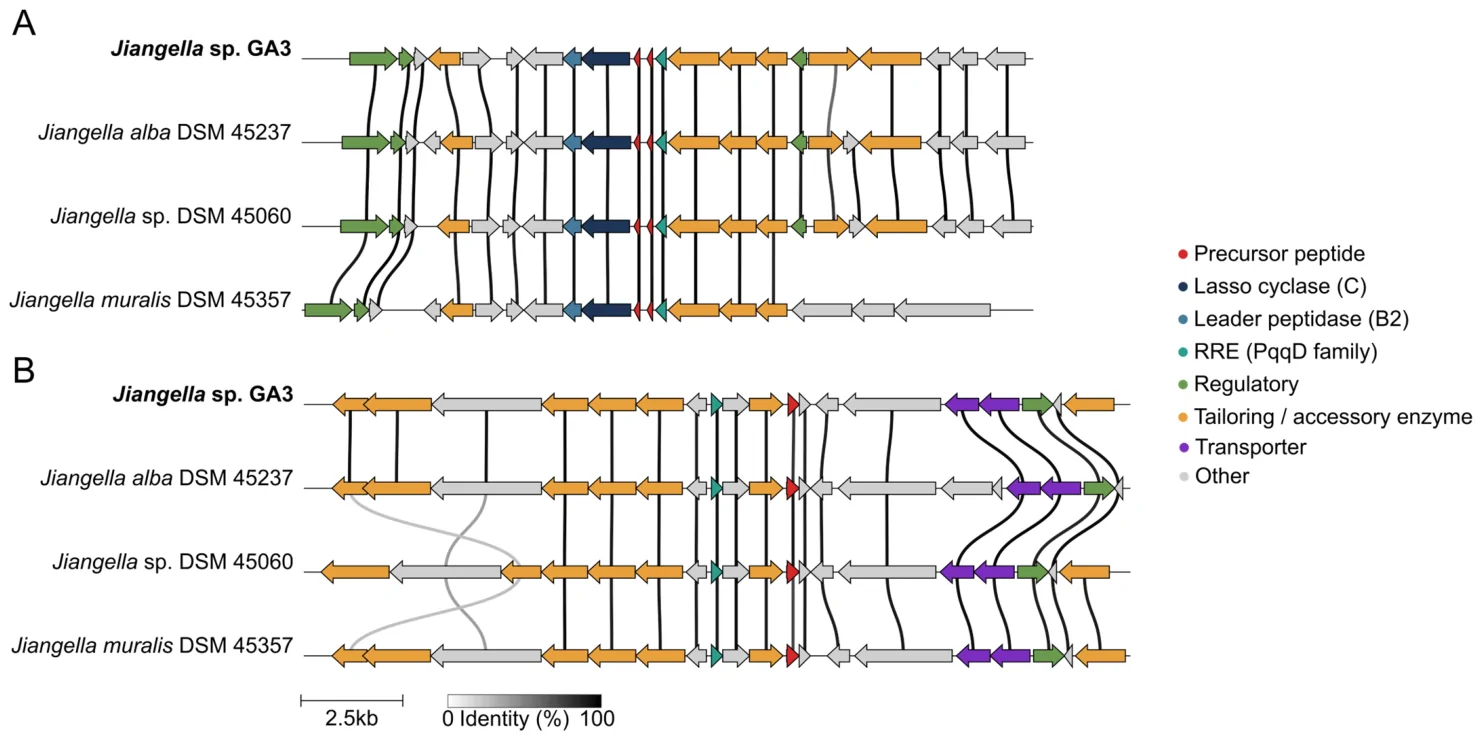
Lasso-peptide (**A**) and RiPP recognition element (RRE)-containing (**B**) loci in *Jiangella* sp. GA3, *Jiangella alba* DSM 45237, *Jiangella* sp. DSM 45060, and *Jiangella muralis* DSM 45357. Loci were reconstructed from the antiSMASH region GenBank files using clinker v0.0.32, oriented in a common direction, and plotted to a common scale; strains are ordered according to the core-gene phylogeny shown in Figure 3. Genes are colored according to their predicted biosynthetic role, and lines connect homologous genes sharing ≥ 30% amino-acid identity, with line shading indicating sequence identity. (**A**) The lasso-peptide locus is shown as an 18-kb segment of the 22.5-kb antiSMASH-predicted region, excluding only flanking sequence lacking coding genes. The locus contains two precursor peptide genes, a B2 leader peptidase, an asparagine synthetase-related lasso cyclase, and a PqqD-family RRE, but no dedicated transporter gene in any of the four strains. Precursor peptide 1 is identical across all four strains, while the maturation enzymes share 93–98% amino-acid identity with their GA3 homologues. (**B**) The RRE-containing locus comprises a DUF6355-family precursor, a PqqD-family RRE, an ABC transporter, and several putative glycosyltransferases and methyltransferases. The J. alba locus forms a separate gene cluster family at a BiG-SCAPE cut-off of 0.3 and groups with the other loci at a cut-off of 0.5.

The most widely distributed families were the hydrogen-cyanide (10/12 genomes), terpene-precursor and mycofactocin (7/12 each), and NRPS-like (6/12). All five RiPP and RiPP-associated clusters of GA3 belonged to families shared within the genus. The lasso-peptide family comprised four strains (GA3, *J. alba*, *J. muralis* and *Jiangella* sp. DSM 45060) at a cut-off of 0.3 and six at 0.5, additionally including *J. asiatica* and *J. gansuensis*. The RiPP recognition element-containing family comprised three strains at 0.3 (GA3, *J. muralis* and *Jiangella* sp. DSM 45060) and six at 0.5, additionally including *J. alba*, *J. asiatica* and *J. gansuensis*. In both families the gene content and order of the locus are conserved across the strains that share it, with homologues of every core biosynthetic gene present in each (Figure 5). Conservation extends to the precursor peptides. The two lasso peptide precursors of GA3 were highly conserved, with the 47-residue precursor being identical and the 44-residue precursor differing by no more than two substitutions among strains sharing the corresponding family (Table S5). The DUF6355 putative precursor of the RiPP recognition element-containing cluster likewise differed by only two substitutions from its homologs over the shared region, and in the annotated position of the start codon. Of the remaining RiPP families, the encapsulin-associated RiPP-like family was present in three strains and the second RiPP-like family in two.

Some functions conserved across the genus were nevertheless encoded by clusters that did not group into a single family: at a cut-off of 0.3, the NAGGN clusters resolved into four families the NI-siderophore clusters into ten, most of them singletons, while every T3PKS region, predicted in all twelve genomes, was assigned to a family of its own (Table S7).

The two strain-specific families of GA3 corresponded to the T3PKS cluster of region GA3_5.r2 and to the uncharacterized cluster (“other”) of region GA3_6.r2 (Table 3). The latter was absent from all other genomes at both cut-offs, and its assignment as a biosynthetic cluster remains uncertain (category IV).

## 3. Discussion

Strain GA3 represents a candidate novel species of the genus *Jiangella*. The highest genome-relatedness values it shares with any type strain fall below the 70% dDDH and 95–96% ANI thresholds that delineate prokaryotic species [23], and both the type-based clustering in TYGS and the core-gene phylogeny recover it as a distinct lineage sister to *J. alba*. Formal description of the species will nonetheless require additional phenotypic and chemotaxonomic characterization, which was beyond the scope of the present study.

Its source is equally unusual: to our knowledge, strain GA3 is the first member of the genus *Jiangella* to be isolated from the biofilm colonizing plastic debris ingested by a loggerhead sea turtle, as previously described species originate from desert and coastal soils, caves, mangrove sediments and plant tissues [16,17,18,19,24]. This extends the range of substrates from which members of the genus have been recovered. However, it does not establish that the strain is adapted to the gut of loggerhead sea turtle, or to the animal-associated plastisphere, as the plastic may have been colonized before ingestion, modified during gut passage, or influenced by rehabilitation conditions.

The GA3 genome is relatively large (7.46 Mb) and encodes an extensive repertoire of transporters, transcriptional regulators, and hydrolytic enzymes, consistent with the broad metabolic versatility reported for many actinobacteria. The interest of this strain lies primarily in the novelty of its clusters rather than their number: only three of the twelve BGCs showed similarity to previously characterized clusters, the single strong match being the T3PKS cluster of region GA3_5.r2, while neither of the two weak matches involved a core biosynthetic gene (Table S4). Consistently, the BiNI of GA3 (865.3) is high relative to values reported for other rare actinobacterial genera, exceeding the genus-level means reported by González-Salazar et al., which ranged from 155.2 to 548.4 [4]. Within *Jiangella*, however, this value is not exceptional: GA3 ranks fourth among twelve genomes, whereas the genus median is 787.6. The elevated biosynthetic divergence therefore appears to be a characteristic of the lineage rather than a unique feature of this strain. If anything, the ranking is favorable to GA3, as three genomes with lower BiNI values contain a high proportion of fragmented BGCs, a factor expected to underestimate the index. Because BiNI also depends on the antiSMASH version used for BGC prediction (v8.0.4 here versus v6.0 in the original study), comparisons with published values should be interpreted as indicative rather than directly quantitative. Of the nine BGCs lacking a MIBiG match, three could be assigned a confident function based on conserved gene content and synteny with characterized systems: the canonical mycofactocin (*mftA–mftF*) locus, a redox-cofactor pathway of central rather than specialized metabolism [22]; the NAGGN cluster, encoding an osmoprotectant compatible solute, a function compatible with the osmotic stress encountered in both saline and arid habitats; and an encapsulin-associated locus encoding the shell protein together with its DyP-type peroxidase cargo, a system indicative of a role in oxidative stress resistance and particularly common among Actinobacteria [25]. Although these loci are detected by antiSMASH because they conform to recognized biosynthetic architectures, they do not encode specialized metabolites in the conventional sense.

Of the remaining six BGCs, none showed similarity to a characterized reference. The second RiPP-like cluster (GA3_7.r1) lacks an unambiguous precursor peptide call, precluding any structural inference. The terpene-precursor cluster contains a polyprenyl synthetase of the FPPS-like family and a phytoene desaturase family protein, but neither a phytoene synthase nor a terpene cyclase, so the pathway cannot be reconstructed, and no scaffold can be inferred. The NRPS-like cluster’s adenylation domains did not yield confident substrate-specificity codes, leaving the peptide backbone composition unresolved. The “other” region (GA3_6.r2) is a borderline case: its assignment rests on two adjacent genes encoding a neocarzinostatin apoprotein domain protein and an HtaA-domain protein, with a DmdR1 binding site predicted with medium confidence immediately upstream, features that in actinobacteria are associated with iron and heme acquisition rather than with secondary metabolism [26,27].

Against this backdrop, the lasso-peptide locus of region GA3_2.r1 and the RiPP recognition element-containing locus of region GA3_14.r1 stand out as the most compelling candidates for detailed analysis, since both the maturation machinery and precursor peptide could be confidently assigned despite the absence of a MIBiG match. The lasso-peptide locus encodes a complete set of maturation enzymes-a lasso cyclase, a B2 leader peptidase, and a PqqD-family RiPP recognition element-together with two distinct precursor peptides of 47 and 44 residues sharing a conserved leader ending in the same GSVRELTL motif, suggesting a small family of related products from a single locus. RODEO placed the leader–core boundary immediately after this motif, consistent with the threonine that occupies the penultimate position of lasso leader peptides and acts as a recognition element for the maturation machinery [28] and predicted in both cases a nine-residue macrolactam closed between the N-terminal alanine and an aspartate at position 9, followed by an aromatic-rich loop. Both features are canonical: small residues are strongly preferred at the first core position of characterized lasso peptides (Gly 62%, Ala 16%), and the acceptor residue falls within the seven- to nine-residue range reported for the lasso macrolactam [29,30]. RODEO nevertheless classified both precursors as negative. This may reflect the limitations of a classifier trained on characterized lasso-peptide families, particularly when applied to divergent or previously uncharacterized sequences. The combination of the conserved precursor architecture, complete maturation machinery, predicted macrolactam, and conservation of both precursors across the GA3/*J. alba*/*J. muralis*/DSM 45060 subclade provides several lines of support for their assignment as candidate lasso-peptide precursors. The RiPP recognition element-containing locus shows a comparable situation: a recognition element, a putative precursor, tailoring enzymes and an ABC export system are all present, which is the canonical composition of a RiPP pathway [29], yet the combination of methylation and glycosylation points to a heavily modified peptide that cannot be assigned to any described class. For neither locus did KnownClusterBlast detect a counterpart among the characterized clusters of the MIBiG repository. In both cases, therefore, the biosynthetic logic can be read in full from the sequence while the nature of the product cannot be predicted from it, which is precisely where sequence-based inference reaches its limit.

Comparative analysis of the predicted BGCs with BiG-SCAPE among strains of the *Jiangella* genus revealed a smaller, conserved repertoire alongside a larger, variable fraction, a pattern consistent with what has been reported for other actinobacterial genera [6,7,8]. The distribution of these families broadly mirrors the phylogeny of the genus (Figure 4), a pattern consistent with vertical inheritance within parts of the genus, together with lineage-specific gain or loss. This repertoire can be partitioned into three components.

The first is a small biosynthetic core shared by most species, within which the hydrogen-cyanide, terpene-precursor, mycofactocin and NRPS-like families are the most broadly conserved.

The second is an intermediate set of families restricted to the subclade formed by GA3, *J. alba*, *J. muralis* and *Jiangella* sp. DSM 45060 and absent from *J. endophytica* and all more distantly related lineages; it includes the lasso-peptide, RiPP recognition element-containing and encapsulin-associated families, none of which corresponds to a characterized biosynthetic pathway. The lasso peptide locus features a 47-residue precursor, which is identical in all four strains, and a second 44-residue precursor differing by a single substitution (Asn to Asp) from that of *J. alba* and by two from that of *Jiangella* sp. DSM 45060. This substitution falls outside the macrolactam ring and is therefore not expected to alter the topology of the product. The putative precursor of the RiPP recognition element-containing cluster is likewise nearly identical to those of the other three strains. Both families are therefore conserved across the subclade down to the precursor peptide itself (Figure 5), and to our knowledge no lasso peptide has so far been isolated from any member of the genus *Jiangella*. This is not unexpected, since lasso peptides are frequently undetectable in their native host under standard culture conditions and heterologous expression is the established route to access such clusters [30].

The third group comprises strain-specific families, which account for approximately three quarters of all families delineated at a BiG-SCAPE cut-off of 0.3. These require cautious interpretation, however, because a family represented by a single genome cannot readily be distinguished from a locus fragmented by assembly breaks or from an artifactual prediction. Only two of the twelve GA3 BGCs formed strain-specific families, a value at the lower end of the range observed across the dataset (two to six per genome) and shared with *J. muralis* and *Jiangella* sp. DSM 45060. One of these is the T3PKS cluster of region GA3_5.r2, three of whose proteins shared 59–65% identity with SrsA, SrsB and SrsC (SGR_470–SGR_472) of the phenolic lipid cluster of *Streptomyces griseus*. The three genes are contiguous and transcribed in the same direction, reproducing the organization of the *S. griseus* operon, so the correspondence rests on conserved gene order as well as on sequence similarity. In *S. griseus,* these enzymes produce alkylresorcinols and alkylquinones that are incorporated into the cell membrane and contribute to membrane integrity and resistance to beta-lactams [31]. Whether the GA3 cluster performs the same function cannot be established from sequence alone, and a UbiA-family prenyltransferase encoded divergently from the operon, with no counterpart in the *S. griseus* cluster, suggests that the product may differ. As noted above, every T3PKS predicted in the genus formed a family of its own, so the strain-specific status of this cluster reflects a genus-wide pattern rather than a peculiarity of GA3. The other strain-specific family corresponds to the “other” region GA3_6.r2 (40.5 kb), whose iron- and heme-related features make its assignment as a biosynthetic cluster uncertain. The absence of these two families from the other genomes might reflect recent acquisition by GA3, although we found no indication of this in either region: no gene annotated as a transposase, integrase, site-specific recombinase or phage-related protein was identified within either region or within 20 kb of its boundaries.

This three-layer picture depends nonetheless on where the similarity threshold is placed. The NAGGN and NI-siderophore functions are conserved across the genus, yet each resolves into several families at a cut-off of 0.3, and the RiPP recognition element-containing cluster of *J. alba* forms a family of its own even though its precursor peptide differs from that of GA3 by only two residues. Family assignment therefore reflects the similarity of entire predicted regions rather than the conservation of the biosynthetic function they encode, and counts obtained at a single cut-off should be read with this in mind. The conservation of the lasso-peptide and RiPP recognition element-containing families across multiple species holds at both cut-offs, the families comprising four and three genomes, respectively, at 0.3 and six at 0.5. This conclusion is therefore not an artifact of the threshold applied.

Overall, these observations qualify the lasso-peptide and RiPP recognition element-containing families as the most promising targets within the genus: they are conserved across a subclade, and therefore unlikely to be artifacts of a single assembly, yet they have no counterpart among characterized clusters. In addition, lasso peptides are known to combine unusual thermal and proteolytic stability with a broad range of reported activities, including antibacterial action, enzyme inhibition and receptor antagonism [29,30], and this combination makes an entirely uncharacterized, genus-conserved family of this class a target worth investigating further.

The tools used gave partly different pictures of the same genome. antiSMASH, rule-based, provided a set of twelve clusters, but its RiPP-like category proved heterogeneous, and its KnownClusterBlast scores, computed over all the genes of a reference cluster, can be driven by genes lying outside the biosynthetic locus. DeepBGC flagged 100 candidate regions, mostly unclassified, and recovered only one antiSMASH RiPP cluster, at low confidence (Table S3). This is expected, as DeepBGC’s predictions are based on a machine-learning classifier, which increases sensitivity but also provides broader, less precisely delimited regions [32,33]. Lacking both a class assignment and defined boundaries, these regions could not be prioritized or compared across genomes, so DeepBGC predictions were treated as an exploratory screen, and the rule-based antiSMASH set was used throughout. BAGEL4 and RODEO performed well on loci with known counterparts but poorly on the two divergent ones. Combining complementary approaches is therefore advisable in taxa for which few reference pathways are available.

GA3 produced no detectable antimicrobial activity against the indicator strains under standard laboratory conditions, although DeepBGC predicted antibacterial activity for the majority of its candidate regions. This is not in itself surprising: only a subset of the predicted clusters would be expected to encode antibacterial metabolites, and many BGCs are transcriptionally silent or only weakly expressed under standard laboratory conditions [34]. Moreover, the agar plug format detects only metabolites that diffuse into the surrounding medium at a sufficient concentration. Furthermore, the bioassay employed here was limited to detecting growth inhibition against three indicator organisms and therefore sampled only a small fraction of the possible biological activities encoded by the genome. The absence of detectable inhibition therefore does not exclude the production of bioactive metabolites. Comparative genome mining cannot resolve this discrepancy because it measures relatedness among BGCs rather than the biological function of their products. Consequently, several explanations remain plausible: the relevant clusters may not have been expressed under the conditions tested; they may have produced metabolites that were inactive against the selected indicator strains or active at concentrations below the detection limit of the assay; alternatively, they may encode compounds with biological activities or target spectra not represented in the present screening.

All analyses presented here are in silico; the structures, modifications and bioactivities inferred for the lasso and RiPP recognition element-containing clusters remain to be verified by confirming precursor expression, isolating and characterizing the products by mass spectrometry and nuclear magnetic resonance spectroscopy, and testing broader bioactivity panels under elicitation conditions. The genus-wide comparison also rests on a limited genome set; only twelve assemblies of acceptable quality are currently available for *Jiangella*, so the classification of families as common, rare, or strain-specific is provisional: it will need revision as more genomes are sequenced, and it remains dependent on the similarity cut-off applied. Finally, although dDDH and OrthoANIu values support assignment of GA3 to a new species, its formal description will require phenotypic and chemotaxonomic characterization [35].

## 4. Materials and Methods

### 4.1. Sampling, Isolation and Identification

A transparent plastic film fragment (sample ID 22146) was recovered non-invasively on 21 August 2024 from the feces of a loggerhead sea turtle (*Caretta caretta*) that had stranded near Ustica Island (Sicily, Italy; approximately 38.71° N, 13.19° E) and was undergoing rehabilitation at the Istituto Zooprofilattico Sperimentale della Sicilia “A. Mirri” (Palermo, Italy).

The fragment was gently rinsed with sterile distilled water to remove loosely attached debris without disrupting the biofilm, transferred to a sterile tube, and preserved at −20 °C. A smaller piece of 1 × 1 cm was cut and resuspended in 1.5 mL of phosphate-buffered saline (PBS). The tube was incubated at 27 °C for 1 h with shaking at 500 rpm; sterile glass beads (0.5 mm in diameter) were then added, and the tube was vortexed for 1 min, with pauses every 15 s, to avoid overheating.

Ten-fold serial dilutions of the resulting suspension were prepared in PBS and 100 µL aliquots were spread-plated, down to 10^−6^, onto Blood Agar (Istituto Zooprofilattico Sperimentale della Sicilia, Palermo, Italy), Marine Agar (MA; Conda, Madrid, Spain) and Starch Casein Agar (soluble starch 10 g L^−1^, casein 0.3 g L^−1^, KNO_3_ 2 g L^−1^, K_2_HPO_4_ 2 g L^−1^, MgSO_4_·7H_2_O 0.05 g L^−1^, CaCO_3_ 0.02 g L^−1^, NaCl 30 g L^−1^, agar 15 g L^−1^), the last of these supplemented with nalidixic acid (25 µg mL^−1^). Plates were incubated aerobically at 30 °C for 7–14 days. Colonies were picked and re-streaked onto Tryptic Soy Agar (TSA; Merck Millipore, Darmstadt, Germany) to obtain axenic cultures. For long-term preservation, the isolate was grown in Tryptic Soy Broth (TSB; Merck Millipore, Darmstadt, Germany) at 30 °C with shaking, and glycerol stocks (25%, v/v) were prepared and stored at −80 °C.

DNA template was obtained directly from a pure culture by colony PCR [36]: one colony was suspended in 100 µL of sterile distilled water, subjected to heat lysis at 99 °C for 15 min, and centrifuged at 10,000× *g* for 15 min. The supernatant was recovered and used as a template. The 16S rRNA gene was amplified with primers F1 (5′-GAGTTTGATCCTGGCTCAG-3′) and R12 (5′-ACGGCTACCTTGTTACGACT-3′) using DreamTaq DNA polymerase (Thermo Fisher Scientific, Waltham, MA, USA), with an initial denaturation of 2 min at 95 °C, 35 cycles of 30 s at 95 °C, 30 s at 56 °C and 90 s at 72 °C, and a final extension of 5 min at 72 °C. Amplification was verified by electrophoresis on a 1% (w/v) agarose gel, and amplicons were Sanger-sequenced with the forward primer only by BMR Genomics s.r.l. (Padova, Italy). The resulting partial sequence was compared against the NCBI database by BLASTn and used for the preliminary assignment of the isolate to the genus *Jiangella*; the near-full-length 16S rRNA gene sequence used for the comparison with type strains (Table S2) was subsequently retrieved from the genome assembly.

### 4.2. Phenotypic Characterization and Antimicrobial Activity

Colony morphology was recorded after 21 days at 30 °C on ISP2 agar (yeast extract 4 g L^−1^, malt extract 10 g L^−1^, dextrose 4 g L^−1^, agar 20 g L^−1^). Growth at different temperatures was assessed on the same medium by streaking from a fresh plate and scoring the extent of biomass development after 14 days at 20, 25, 30, 37 and 50 °C on a semi-quantitative scale (+++, abundant growth; ++, moderate growth; +, scant growth; −, no growth).

Antimicrobial activity was screened by an agar plug diffusion assay. Strain GA3 was grown for 6–7 days at 30 °C on MA, mannitol-soya flour agar (mannitol 20 g L^−1^, soya flour 20 g L^−1^, agar 20 g L^−1^), Czapek’s agar (sucrose 30 g L^−1^, NaNO_3_ 3 g L^−1^, K_2_HPO_4_ 1 g L^−1^, MgSO_4_·7H_2_O 0.5 g L^−1^, KCl 0.5 g L^−1^, FeSO_4_·7H_2_O 0.01 g L^−1^, agar 15 g L^−1^) and R5A agar (K_2_SO_4_ 0.25 g L^−1^, MgCl_2_·6H_2_O 10.12 g L^−1^, glucose 10 g L^−1^, casamino acids 0.1 g L^−1^, yeast extract 5 g L^−1^, MOPS 21 g L^−1^, agar 15 g L^−1^, and 2 mL L^−1^ of a trace element solution containing ZnCl_2_ 0.04 g L^−1^, FeCl_3_·6H_2_O 0.2 g L^−1^, CuCl_2_·2H_2_O 0.01 g L^−1^, MnCl_2_·4H_2_O 0.01 g L^−1^, Na_2_B_4_O_7_·10H_2_O 0.01 g L^−1^ and (NH_4_)_6_Mo_7_O_24_·4H_2_O 0.01 g L^−1^; adjusted to pH 6.8 with KOH) and agar plugs (6 mm in diameter) were transferred onto TSA plates previously inoculated with the indicator strains *Kocuria rhizophila* ATCC 9341, *Staphylococcus aureus* ATCC 25923 and *Escherichia coli* ATCC 25922. Plates were incubated at 30 °C for 24 h. Inhibition was scored as any visible absence of indicator growth extending beyond the edge of the plug, and, where present, the inhibition zone was measured as its diameter (mm). Two independent biological replicates were performed.

### 4.3. DNA Extraction

Genomic DNA of strain GA3 was extracted from a 25 mL culture grown in R5A medium for 3 days at 30 °C using a salting-out procedure, adapted from Kieser et al. [37]. Briefly, the cell pellet was resuspended in SET buffer (75 mM NaCl, 25 mM EDTA, pH 8.0, 20 mM Tris-HCl pH 7.5) and lysed with lysozyme (1 mg/mL, 37 °C, 1 h), followed by SDS and proteinase K (0.5 mg/mL, 55 °C, 2 h). Proteins were precipitated with 5 M NaCl and removed by chloroform extraction. Genomic DNA was precipitated with isopropanol, spooled, washed with 70% ethanol, and dissolved in sterile distilled water. DNA purity and concentration were assessed with a NanoDrop spectrophotometer, and integrity by 0.8% agarose gel electrophoresis.

### 4.4. Whole-Genome Sequencing and Genome Assembly

Whole-genome sequencing and primary bioinformatic analysis were performed by mBioWorks ApS (Copenhagen, Denmark) based on an internal pipeline. Libraries were sequenced on an Illumina NovaSeq 6000 platform (paired-end, 2 × 150 bp; ≥2 Gb per sample). Sequencing yielded 7,802,320 read pairs of 150 bp. Adapter removal and quality trimming were performed with fastp v0.24.0, retaining 7,749,691 read pairs and corresponding to an average genome coverage of 287×. Reads were assembled de novo with Unicycler v0.5.0. Contigs shorter than 1 kb were removed prior to annotation and BGC prediction. Assembly metrics were computed with QUAST, and genome completeness and contamination were assessed with CheckM. The genome was annotated with the NCBI Prokaryotic Genome Annotation Pipeline (PGAP) [38]. General functional categories were derived from the NCBI PGAP annotation of the GA3 genome by grouping predicted protein products into functional classes; counts refer to the number of protein-coding genes assigned to each class.

### 4.5. Phylogenomic Analysis and Species Delimitation

Taxonomic placement was first assessed with GTDB-Tk v2.7.2 [39], against GTDB release R226. Genome-based taxonomy was then refined using the Type (Strain) Genome Server (TYGS; https://tygs.dsmz.de; accessed on 13 July 2026) [40,41,42], with nomenclatural information retrieved from the List of Prokaryotic names with Standing in Nomenclature (LPSN) [40,41]. All TYGS and LPSN analyses were performed on 10 July 2026. Within TYGS, the closest type strains were identified both from genome-wide MASH distances [43] and from the 16S rRNA gene, extracted with RNAmmer [44] and compared by BLAST+ [45]. Intergenomic distances were computed with the Genome BLAST Distance Phylogeny approach (GBDP; algorithm ‘trimming’, distance formula d5) over 100 replicates [46], and digital DNA–DNA hybridization (dDDH) values with confidence intervals were obtained with GGDC 4.0 under recommended settings [41,46]. Type-based species delimitation was carried out using a 70% dDDH radius around each type strain [42]. A concatenated core-gene maximum-likelihood tree was reconstructed with autoMLST2 (IQ-TREE) [47], with *Klenkia taihuensis* as the outgroup. The tree was visualized and annotated in iTOL [48]. Average nucleotide identity (ANI) was additionally calculated with the OrthoANIu tool [49] through EzBioCloud (www.ezbiocloud.net/tools/ani; accessed on 13 July 2026). The circular genome map was drawn with Proksee (https://proksee.ca; accessed on 29 July 2026), including genomic features such as coding sequences, tRNA and rRNA genes, GC content, GC skew and the predicted BGC regions; these were computed within Proksee over a 10,000 bp window with a 1000 bp step [50].

### 4.6. Bioinformatic Prediction of Biosynthetic Gene Clusters

BGCs were predicted with antiSMASH v8.0.4 under relaxed strictness [32] and with DeepBGC (default parameters, detection score ≥ 0.5) [33]. Similarity to characterized pathways was assessed from the antiSMASH KnownClusterBlast comparison against the MIBiG repository [51]. Biosynthetic novelty was quantified as the BiNI of González-Salazar et al. [4]. The predicted regions were grouped into four categories: canonical specialized metabolite clusters (I), RiPP candidate pathways (II), loci associated with cofactor, metabolic, or stress-related functions rather than canonical specialized metabolism (III), and predictions for which no defined biosynthetic class could be assigned (IV). Each antiSMASH job was submitted to the BiG-FAM platform (accessed on 5 July 2026), which returns the distance (d) of every predicted region to the closest gene cluster family in the database; the index was calculated as the sum of the distances exceeding the novelty threshold of 900, divided by the total number of regions predicted in that genome. Ribosomally synthesized and post-translationally modified peptide (RiPP) clusters were examined further with BAGEL4 (accessed on 25 July 2026) [52], and with RODEO [53] (peptide type ‘lasso’, default parameters, window of ±8 coding sequences) to score the lasso precursors and predict their leader–core boundaries and macrolactam topology.

### 4.7. Comparative Analysis of Biosynthetic Gene Clusters Across the Genus

All *Jiangella* genome assemblies available in NCBI RefSeq (accessed 13 July 2026) were retrieved (*n* = 14; Supplementary Table S8). Draft genomes consisting of more than 300 contigs were omitted from downstream analyses due to assembly quality constraints (e.g., *J. rhizosphaerae* NEAU-YY265ᵀ), following Adamek et al. [7]; when a species was represented by more than one assembly, the more contiguous assembly was retained. The final dataset comprised 12 genomes: strain GA3, the type strains of ten validly published *Jiangella* species, and *Jiangella* sp. DSM 45060. Reference genomes were analyzed with antiSMASH v8.0.4 using the same settings as applied to GA3, yielding 124 BGC regions in total. BiNI was calculated for each reference genome as described for strain GA3, using the same antiSMASH version and detection strictness, so that values are directly comparable across the twelve genomes. Regions were compared using BiG-SCAPE v2.0.3 [54] with the Pfam 38.2 database, binned both by antiSMASH class and as a combined (“mix”) dataset, with singletons retained. To account for sequence divergence among homologous clusters, GCFs were delineated at two distance cut-offs: 0.3 (the tool default, grouping closely related clusters into smaller families) and 0.5 (merging more divergent homologs), an approach also adopted in comparative analyses of other actinobacterial genera [8]. Unless otherwise stated, all reported GCF assignments correspond to the 0.3 cut-off. Reference clusters from the MIBiG repository were not included, so that GCF assignments reflect relatedness within the genus rather than similarity to characterized pathways. GCFs were classified as operational categories: common (present in ≥ 4 strains), rare (2–3 strains) or strain-specific (present in a single strain), as described in Adamek et al. [7]. Precursor peptides of the lasso-peptide, RiPP recognition element-containing, and mycofactocin families were extracted from the GenBank files of the corresponding antiSMASH regions and compared with those of strain GA3 by pairwise alignment. The lasso-peptide and RiPP recognition element-containing loci of strains representing each family were visualized and compared using clinker v0.0.32 [55] from the corresponding antiSMASH region GenBank files, and homologous genes were linked when they shared at least 30% amino acid identity. Loci were oriented in a common direction, and genes were colored according to their assigned biosynthetic role.

## 5. Conclusions

Strain GA3 represents a candidate novel species of the genus *Jiangella* and, to our knowledge, the first member of the genus to be recovered from plastic debris associated with a marine animal. Its genome encodes twelve BGCs, a number typical of the genus, yet only one of them has a characterized counterpart, and its BiNI places the repertoire far from the families catalogued to date. Comparing these clusters with those of the eleven other *Jiangella* genomes available showed that the distribution of several BGC families is consistent with vertical inheritance within parts of the genus, together with lineage-specific gain or loss, and that the loci without characterized counterparts are conserved across entire lineages rather than confined to single strains. Among them, the lasso-peptide family is the clearest starting point for experimental work: it is conserved across four species at a cut-off of 0.3 and six at 0.5, and no lasso peptide has yet been isolated from any member of the genus. The clusters described here remain computational predictions, as no metabolite was isolated and no antimicrobial activity was detected under the conditions tested. Accessing and characterizing their products will require dedicated activation strategies, untargeted metabolomic profiling, mass spectrometry and nuclear magnetic resonance spectroscopy, together with broader bioactivity screening. Together with the deposited genome, these results provide both a defined taxonomic entity and a prioritized set of targets for the chemical exploration of an underexplored actinobacterial lineage.

## Figures and Tables

**Table 1:** Genome assembly and annotation summary for *Jiangella* sp.

Features	GA3
Genome size (bp)	7,462,732
G+C content (mol%)	72.81
No. of contigs	27
Largest contig (bp)	1,369,243
N50 (bp)	886,138
N90 (bp)	230,876
L50	4
L90	9
N’s per 100 kbp	0.00
N’s	0
Completeness (CheckM, %)	100.00
Contamination (CheckM, %)	0.59
Coverage	287×
Total genes	6815
Protein-coding genes (CDS)	6763
tRNA genes	46
rRNA genes	3

**Table 2:** Digital DNA–DNA hybridization (dDDH, formula d4) and OrthoANIu values between strain GA3 and the type strains of the validly published species of the genus *Jiangella*.

Type Strain	dDDH d4 (%)	OrthoANIu (%)
*J. alba* DSM 45237^T^	57.8	94.65
*J. muralis* DSM 45357^T^	51.0	93.23
*J. endophytica* KE2-3^T^	37.7	89.35
*J. alkaliphila* DSM 45079^T^	36.1	88.62
*J. rhizosphaerae* NEAU-YY265^T^	35.8	88.31
*J. aurantiaca* 8K307^T^	35.1	88.04
*J. anatolica* GTF31^T^	34.6	88.08
*J. mangrovi* DSM 102122^T^	31.1	86.34
*J. asiatica* 5K138^T^	24.9	81.76

**Table 3:** Biosynthetic gene clusters predicted in the genome of *Jiangella* sp. GA3 by antiSMASH. Category: I, canonical specialized metabolite BGC; II, RiPP candidate pathway; III, cofactor-, metabolic- or stress-related locus; IV, prediction for which no defined biosynthetic class could be assigned. ND, not determined (no MIBiG hit).

Region	Type *	Category	Length (bp)	Similar to	KnownClusterBlast Confidence	MIBiG Accession ID
GA3_1.r1	NI-siderophore	I	33.345	Schizokinen	Low	BGC0002683
GA3_1.r2	Hydrogen-cyanide	I	12.801	Aborycin	Low	BGC0002285
GA3_2.r1	Lasso-peptide	II	22.538	ND	ND	
GA3_2.r2	Redox-cofactor	III	29.482	ND	ND	
GA3_5.r1	Terpene-precursor	I	21.100	ND	ND	
GA3_5.r2	T3PKS	I	41.079	2-methoxy-5-methyl-6-(13-methyltetradecyl)-1,4-benzoquinone/2-methoxy-5-methyl-6-(13-methyltetradecyl)phenol	High	BGC0000282
GA3_6.r1	NAGGN	III	18.205	ND	ND	
GA3_6.r2	Other	IV	40.476	ND	ND	
GA3_6.r3	NRPS-like	I	42.540	ND	ND	
GA3_7.r1	RiPP-like	IV	11.496	ND	ND	
GA3_8.r1	RiPP-like	III	10.800	ND	ND	
GA3_14.r1	RiPP recognition element-containing	II	20.281	ND	ND	

* Product classes are reported as assigned by antiSMASH: NI-siderophore, non-ribosomal peptide synthetase (NRPS)-independent siderophore; T3PKS, type III polyketide synthase; NAGGN, *N*-acetylglutaminylglutamine amide; NRPS-like, non-ribosomal peptide synthetase-like; RiPP-like, ribosomally synthesized and post-translationally modified peptide-like.

## Data Availability

The draft genome sequence of *Jiangella* sp. GA3 has been deposited at DDBJ/ENA/GenBank under BioProject PRJNA1508417 and BioSample SAMN62232622, with assembly accession GCA_060210745.1. This Whole Genome Shotgun project has been deposited under the accession JCBRNP000000000; the version described in this paper is JCBRNP010000000. The genome assemblies used in the comparative analysis are publicly available in NCBI RefSeq, with accession numbers listed in Table S8. All other data supporting the findings of this study are included in the article and its Supplementary Materials.

## Supplementary Materials

The following supporting information can be downloaded at https://www.mdpi.com/article/10.3390/md24090319/s1, Table S1: Functional categories and mobile genetic elements encoded in the GA3 genome; Table S2: Closest 16S rRNA gene hits (type strains) for strain GA3; Table S3: Complete set of biosynthetic gene cluster predictions generated by DeepBGC (*n* = 100); Table S4: Protein-level similarity between the BGCs of strain GA3 and characterized clusters of the MIBiG repository; Table S5: Precursor peptides of the lasso-peptide, RiPP recognition element-containing and mycofactocin clusters; Table S6: Biosynthetic Novelty Index of the twelve Jiangella genomes; Table S7: Assignment of the 124 antiSMASH regions to gene cluster families at cut-offs of 0.3 and 0.5; Table S8: Jiangella genome assemblies retrieved from NCBI RefSeq and used in the comparative analysis.

## Abbreviations

The following abbreviations are used in this manuscript:

ANI	average nucleotide identity
BGC	biosynthetic gene cluster
BiNI	Biosynthetic Novelty Index
dDDH	digital DNA–DNA hybridization
DyP	dye-decolorizing peroxidase
FPPS	farnesyl pyrophosphate synthase
GCF	gene cluster family
MA	Marine Agar
MIBiG	Minimum Information about a Biosynthetic Gene cluster
NAGGN	N-acetylglutaminylglutamine amide
NI-siderophore	NRPS-independent siderophore
NRPS	non-ribosomal peptide synthetase
PBS	phosphate-buffered saline
RiPP	ribosomally synthesized and post-translationally modified peptide
RRE	RiPP recognition element
SAM	S-adenosylmethionine
T3PKS	type III polyketide synthase
TFBS	transcription factor binding site
TSA	tryptic soy agar
