# Supplementary material for: Comparative Genome Mining of Jiangella sp. GA3 from Turtle-Ingested Plastic Reveals Conserved Biosynthetic Gene Clusters
Source: Mar Drugs. 2026 Sep 10;24(9):319. doi: 10.3390/md24090319 (PMC13608877; doi:10.3390/md24090319)
Supplement: Supplementary file 1 [file marinedrugs-24-00319-s001.zip › marinedrugs-4517039-supplementary.pdf]

1. Supplementary material

Table S1. Functional categories and mobile genetic elements encoded in the genome of *Jiangella* sp. GA3. Counts refer to protein-coding genes whose product, as assigned by the NCBI Prokaryotic Genome Annotation Pipeline (PGAP), matched each category; insertion sequence families are those assigned by the same annotation.

Category	No. of genes
**General functional categories**	
Protein-coding genes (CDS)	6,763
Hypothetical proteins	968
ABC transporter components	706
- permeases	342
- ATP-binding proteins	172
- substrate-binding proteins	164
MFS transporters	102
Transcriptional regulators	472
Sigma factors	74
Histidine kinases	44
Response regulators	58
Glycoside hydrolases	53
Glycosyltransferases	45
Peptidases and proteases	169
Esterases and lipases	11
α/β-hydrolase fold proteins	62
Cytochrome P450	5
NADH:quinone oxidoreductase subunits	13
Cytochrome bc1 subunits	5
Terminal oxidase subunits	10
ATP synthase subunits	8
Thioredoxins and peroxiredoxins	11
Mycothiol biosynthesis	7
Trehalose biosynthesis	4
Pupylation–proteasome system	6
Catalase	1
Superoxide dismutase	1
Nitrogenase	0
Urease	0
Ectoine biosynthesis	0
Type VII secretion	0
**Mobile genetic elements**	
Transposases	17
- intact	9
- annotated as pseudogenes	8
- IS110 family	5
- IS982 family	2
- IS3 family	1
- IS481 family	1
- IS630 family	1
- IS5/IS1182 family	1
- family not assigned	6
Integrases	11
- intact	10
- annotated as pseudogene	1
Phage structural genes	9
Conjugative transfer genes	4

Table S2. Closest 16S rRNA gene hits (type strains) for strain GA3, with NCBI RefSeq accession, query coverage and percentage identity.

Type strain	NCBI accession	Query coverage (%)	16S identity (%)
Jiangella alba YIM 61503^T	NR_116547	99	99.80
Jiangella asiatica 5K138^T	NR_179916	98	99.26
Jiangella endophytica KE2-3^T	NR_171463	99	99.14
Jiangella aurantiaca 8K307^T	NR_179919	97	98.98
Jiangella rhizosphaerae NEAU-YY265^T	NR_171511	99	98.94
Jiangella anatolica GTF31^T	NR_164947	98	98.93
Jiangella mangrovi 3SM4-07^T	NR_136795	97	98.58
Jiangella gansuensis DSM 44835^T	NR_043041	98	98.46
Jiangella ureilytica KC603^T	NR_179915	97	98.31
Haloactinopolyspora alkaliphila EGI 80088^T	NR_133755	99	97.49

Table S3. Complete set of biosynthetic gene cluster predictions generated by DeepBGC in the genome of *Jiangella* sp. GA3 (n = 100), listing for each candidate region the contig, genomic coordinates, size, number of predicted proteins, DeepBGC detection score, predicted product class and predicted activity, and the overlapping antiSMASH-predicted BGC where applicable. Regions for which no product class exceeded the classification threshold are reported as unassigned.

Contig	Location (bp)	Length (bp)	Proteins	DeepBGC score	Predicted class	Predicted activity	antiSMASH BGC
GA3_1	49,373–49,964	591	1	0.52	unassigned	—	—
GA3_1	82,723–87,628	4,905	2	0.56	unassigned	antibacterial	—
GA3_1	198,484–201,512	3,028	4	0.79	Polyketide	—	—
GA3_1	239,984–241,478	1,494	1	0.53	unassigned	antibacterial	—
GA3_1	259,452–263,204	3,752	5	0.67	unassigned	—	—
GA3_1	391,509–393,423	1,914	1	0.50	unassigned	antibacterial	—
GA3_1	409,401–423,719	14,318	14	0.65	unassigned	antibacterial	NI-siderophore
GA3_1	431,447–446,959	15,512	13	0.58	Other	—	NI-siderophore
GA3_1	485,270–493,080	7,810	8	0.57	RiPP	antibacterial	—
GA3_1	495,641–497,206	1,565	2	0.54	RiPP	antibacterial	—
GA3_1	504,427–523,792	19,365	18	0.64	unassigned	—	—
GA3_1	530,662–538,465	7,803	5	0.64	unassigned	antibacterial	—
GA3_1	556,009–584,024	28,015	25	0.66	RiPP	antibacterial	—
GA3_1	751,329–766,834	15,505	17	0.80	unassigned	cytotoxic	—
GA3_1	988,212–999,116	10,904	11	0.62	unassigned	antibacterial	—
GA3_1	999,839–1,001,095	1,256	2	0.54	unassigned	antibacterial	—
GA3_1	1,005,318–1,005,654	336	1	0.52	unassigned	antibacterial	—
GA3_1	1,088,189–1,088,756	567	1	0.54	unassigned	antibacterial	—
GA3_1	1,091,724–1,181,949	90,225	76	0.90	Saccharide	—	—
GA3_1	1,358,929–1,367,367	8,438	9	0.60	unassigned	antibacterial	—

GA3_1	1,369,051–1,369,243	192	1	0.52	unassigned	antibacterial	—
GA3_2	15,439–15,895	456	1	0.51	RiPP	antibacterial	lassopeptide
GA3_2	17,714–17,978	264	1	0.56	unassigned	antibacterial	lassopeptide
GA3_2	88,414–90,719	2,305	3	0.73	unassigned	—	—
GA3_2	164,954–167,144	2,190	2	0.55	Polyketide	antibacterial	—
GA3_2	169,264–181,421	12,157	13	0.92	Saccharide	—	—
GA3_2	185,347–188,650	3,303	1	0.55	RiPP	antibacterial	—
GA3_2	239,178–240,318	1,140	1	0.54	Polyketide	antibacterial	—
GA3_2	240,468–257,776	17,308	17	0.75	unassigned	antibacterial	—
GA3_2	262,394–263,970	1,576	3	0.56	Polyketide	antibacterial	—
GA3_2	267,359–271,093	3,734	5	0.60	unassigned	antibacterial	—
GA3_2	272,798–273,803	1,005	1	0.53	NRP	inhibitor	—
GA3_2	417,563–423,392	5,829	3	0.75	Other	cytotoxic	—
GA3_2	465,493–492,239	26,746	20	0.78	unassigned	cytotoxic	—
GA3_2	506,840–507,839	999	1	0.54	RiPP	antibacterial	—
GA3_2	558,375–560,134	1,759	2	0.57	RiPP	antibacterial	—
GA3_3	9,128–10,472	1,344	3	0.54	unassigned	antibacterial	—
GA3_3	18,921–19,332	411	1	0.51	unassigned	antibacterial	—
GA3_3	72,247–73,828	1,581	1	0.52	Polyketide	antibacterial	—
GA3_3	288,155–289,862	1,707	1	0.54	unassigned	—	—
GA3_3	315,235–316,560	1,325	2	0.56	unassigned	—	—
GA3_3	371,729–380,113	8,384	7	0.57	RiPP	antibacterial	—
GA3_3	446,791–452,013	5,222	2	0.62	Other	antibacterial	—
GA3_3	742,434–771,556	29,122	40	0.75	unassigned	—	—
GA3_3	979,585–980,335	750	1	0.57	unassigned	antibacterial	—
GA3_3	980,999–1,021,150	40,151	33	0.68	unassigned	antibacterial	—
GA3_3	1,022,159–1,026,550	4,391	5	0.53	unassigned	antibacterial	—
GA3_4	41,191–42,999	1,808	2	0.56	Other	—	—
GA3_4	44,383–46,545	2,162	2	0.56	unassigned	antibacterial	—
GA3_4	492,257–497,268	5,011	4	0.60	RiPP	antibacterial	—
GA3_4	503,604–522,046	18,442	21	0.77	unassigned	antibacterial	—
GA3_4	530,030–541,343	11,313	11	0.63	Other	—	—
GA3_4	682,881–686,770	3,889	3	0.53	unassigned	antibacterial	—
GA3_4	711,526–715,675	4,149	5	0.65	unassigned	antibacterial	—
GA3_4	717,907–730,892	12,985	8	0.58	Polyketide	antibacterial	—
GA3_5	307,119–308,354	1,235	2	0.54	unassigned	antibacterial	—
GA3_5	308,972–309,335	363	1	0.60	unassigned	antibacterial	—
GA3_5	313,561–319,965	6,404	5	0.64	unassigned	antibacterial	—
GA3_5	477,249–478,988	1,739	2	0.54	RiPP	—	—
GA3_5	486,484–487,429	945	1	0.54	unassigned	antibacterial	terpene-precursor
GA3_5	489,272–490,355	1,083	1	0.54	Saccharide-Terpene	—	terpene-precursor
GA3_5	493,165–494,266	1,101	1	0.58	Polyketide-Terpene	antibacterial	terpene-precursor
GA3_5	495,102–500,551	5,449	7	0.62	Polyketide	antibacterial	terpene-precursor
GA3_5	644,329–670,677	26,348	20	0.90	RiPP	—	—

GA3_5	670,999–672,250	1,251	1	0.53	unassigned	antibacterial	—
GA3_5	679,757–708,536	28,779	24	0.73	unassigned	—	T3PKS
GA3_5	710,232–724,771	14,539	8	0.68	unassigned	antibacterial	T3PKS
GA3_5	798,294–799,284	990	1	0.53	unassigned	antibacterial	—
GA3_5	816,129–818,974	2,845	3	0.58	unassigned	antibacterial	—
GA3_5	822,850–824,482	1,632	1	0.52	unassigned	antibacterial	—
GA3_6	146,598–147,891	1,293	1	0.50	unassigned	antibacterial	—
GA3_6	245,007–316,744	71,737	67	0.90	Polyketide	antibacterial	other
GA3_6	332,039–344,276	12,237	9	0.59	RiPP	—	—
GA3_6	396,182–397,331	1,149	1	0.51	unassigned	antibacterial	—
GA3_6	398,356–405,775	7,419	7	0.63	unassigned	antibacterial	—
GA3_6	407,300–407,750	450	1	0.53	unassigned	antibacterial	—
GA3_6	408,400–408,880	480	1	0.59	unassigned	antibacterial	—
GA3_7	100,171–100,603	432	1	0.63	unassigned	antibacterial	—
GA3_7	101,225–102,284	1,059	2	0.51	unassigned	—	—
GA3_7	223,347–228,332	4,985	6	0.62	NRP	antibacterial	—
GA3_7	231,521–243,025	11,504	9	0.59	Saccharide	antibacterial	—
GA3_7	244,607–246,343	1,736	2	0.56	unassigned	—	—
GA3_7	331,442–331,883	441	1	0.53	unassigned	antibacterial	—
GA3_8	87,909–88,995	1,086	1	0.53	unassigned	antibacterial	—
GA3_8	92,398–97,129	4,731	5	0.54	Polyketide	inhibitor	RiPP-like
GA3_8	153,494–154,034	540	1	0.54	unassigned	antibacterial	—
GA3_9	36,169–47,955	11,786	15	0.76	unassigned	—	—
GA3_9	228,085–230,876	2,791	2	0.56	unassigned	antibacterial	—
GA3_10	107,579–208,017	100,438	88	0.84	unassigned	—	—
GA3_11	49,608–69,788	20,180	16	0.64	Other	—	—
GA3_11	131,906–147,432	15,526	20	0.71	unassigned	antibacterial	—
GA3_11	149,137–154,675	5,538	7	0.53	RiPP	antibacterial	—
GA3_11	162,850–163,733	883	2	0.56	unassigned	antibacterial	—
GA3_12	1,140–5,768	4,628	7	0.67	unassigned	antibacterial	—
GA3_12	6,630–8,598	1,968	1	0.50	RiPP	antibacterial	—
GA3_12	11,190–14,169	2,979	1	0.50	unassigned	antibacterial	—
GA3_14	1,529–50,969	49,440	46	0.95	Saccharide	—	RRE-containing
GA3_15	7,186–10,069	2,883	1	0.55	Other	antibacterial	—
GA3_26	0–985	985	1	0.60	Polyketide	antibacterial	—
GA3_27	0–627	627	1	0.52	unassigned	antibacterial	—

Table S4. Protein-level detail of the similarity between the biosynthetic gene clusters of *Jiangella* sp. GA3 and characterized clusters of the MIBiG repository. Data are the KnownClusterBlast output of antiSMASH v8.0.4 for the three regions for which a most similar known cluster was reported (Table 3); the similarity confidence assigned by antiSMASH is given for each. MIBiG accessions correspond to BGC0000282 (2-methoxy-5-methyl-6-(13-methyltetradecyl)-1,4-benzoquinone / phenol, type III PKS), BGC0002683 (schizokinen) and BGC0002285 (aborycin); cumulative BLAST scores were 981, 824 and 1062, respectively. For the remaining nine regions antiSMASH reported no most similar known cluster. Percentage identity and query coverage refer to the pairwise alignment of each protein with its homologue

in the reference cluster; note that only region GA3_5.r2 includes a hit to a core biosynthetic gene.

Region	Confidence	MIBiG cluster	GA3 locus tag	Annotated function	Subject gene	Identity (%)	Coverage (%)	E-value
GA3_5.r2	High	BGC0000282	pgaptmp_005420	type III polyketide synthase (core biosynthetic gene)	SGR_472 (SrsA)	59	99.7	5.46e-142
GA3_5.r2	High	BGC0000282	pgaptmp_005419	isoprenylcysteine carboxyl methyltransferase family protein	SGR_471 (SrsB)	65	96.0	1.75e-72
GA3_5.r2	High	BGC0000282	pgaptmp_005418	NAD(P)/FAD-dependent oxidoreductase	SGR_470 (SrsC)	61	99.1	1.65e-123
GA3_1.r1	Low	BGC0002683	pgaptmp_000404	lysine N(6)-hydroxylase / L-ornithine N(5)-oxygenase family protein	BAB72350.1	50	98.6	4.86e-143
GA3_1.r1	Low	BGC0002683	pgaptmp_000408	diaminobutyrate–2-oxoglutarate transaminase family protein	BAB72354.1	52	93.7	2.38e-139
GA3_1.r2	Low	BGC0002285	pgaptmp_001226	proline racemase family protein	BIU87_04120	72	100.0	1.46e-178
GA3_1.r2	Low	BGC0002285	pgaptmp_001227	dihydrodipicolinate synthase family protein	BIU87_04115	63	92.6	2.66e-131
GA3_1.r2	Low	BGC0002285	pgaptmp_001225	GntR family transcriptional regulator	BIU87_04125	47	95.5	3.48e-60

Table S5. Precursor peptides of the lasso-peptide, RiPP recognition element-containing and mycofactocin clusters of *Jiangella* sp. GA3 and of the corresponding clusters in the other members of their gene cluster families.

Cluster / precursor	Organism	Locus tag	Length (aa)	Sequence
Lasso peptide precursor 1	*Jiangella* sp. GA3	pgaptmp_001816	47	MAYQPPVLEEVGSVRELTLAQGGQGSSDQ FLWFKWGNDKPPGGGGLS
Lasso peptide precursor 2	*Jiangella* sp. GA3	pgaptmp_001817	44	MAYEAPTLEEIGSVRELTLAASGRGRSDQV QWFRYNNDPGGVLS
Lasso peptide precursor 1	*Jiangella alba* DSM 45237	BLV02_RS35955	47	MAYQPPVLEEVGSVRELTLAQGGQGSSDQ FLWFKWGNDKPPGGGGLS
Lasso peptide precursor 2	*Jiangella alba* DSM 45237	BLV02_RS35950	44	MAYEAPTLEEIGSVRELTLAASGRGRSDQV QWFRYDNDPGGVLS
Lasso peptide precursor 1	*Jiangella muralis* DSM 45357	ACW62_RS34385	47	MAYQPPVLEEVGSVRELTLAQGGQGSSDQ FLWFKWGNDKPPGGGGLS
Lasso peptide precursor 2	*Jiangella muralis* DSM 45357	ACW62_RS34390	44	MAYEAPTLEEIGSVRELTLAASGRGRSDQV QWFRYNNDPGGVLS
Lasso peptide precursor 1	*Jiangella* sp. DSM 45060	BLU82_RS34425	47	MAYQPPVLEEVGSVRELTLAQGGQGSSDQ FLWFKWGNDKPPGGGGLS
Lasso peptide precursor 2	*Jiangella* sp. DSM 45060	BLU82_RS34430	44	MAYEAPTLEEIGTVRELTLAASGRGRSDQV QWFRYDNDPGGVLS
RRE-containing precursor (DUF6355)	*Jiangella* sp. GA3	pgaptmp_001755	92	MAFAGGLVVGGAAASAQAVPPPCGFYEV GGGFLNDDMYNHCGDHPVLVTMVGLGIY NQYTACLAPGVHDLNEIFNNPVVDAYSEG EPCSDPE
RRE-containing precursor (DUF6355)	*Jiangella alba* DSM 45237	BLV02_RS28390	103	MNKGKAAMLAAVALAGGVVAGGAAASAQ AVPPPCGFYEVGGGFLNDDMYNHCGDHP VWVTMVGLGIYNQYTACLAPGVHDLNEIF NNPVLDAYSEGEPCSDPE
RRE-containing precursor (DUF6355)	*Jiangella muralis* DSM 45357	ACW62_RS31015	103	MKWGKVAFTATAAVFAGGLLAGGVAASAQ AVPPPCGFYEVGGIFHDDMYNHCGDHPV LVTMVGYGMYNQYTACLAPGVHDLNEIFN AAVIDAYSEGERCSDPE
RRE-containing precursor (DUF6355)	*Jiangella* sp. DSM 45060	BLU82_RS24405	104	MTWGKIARSAAAVALAGGVVAGGAAASAQ AVPPPCGFYEVGGGFLNDDMYNHCGDHP VWVTMVGLGLYNQYTACLAPGVHDLNEIF NNPVLDAYSEGEPCSDPE
Mycofactocin precursor MftA	*Jiangella* sp. GA3	pgaptmp_002173	34	MTQTDQTAVTVPDDLVEDDSLVEEVSIDG MCGVY

RODEO output for the two lasso precursors of *Jiangella* sp. GA3: precursor 1 (pgaptmp_001816), heuristic score 10, predicted core AQGGQGSSDQFLWFKWGNDKPPGGGGLS, calculated lasso mass 2859 Da; precursor 2 (pgaptmp_001817), heuristic score 9, predicted core AASGRGRSDQVQWFRYNNDPGGVLS, calculated lasso mass 2718 Da. Both were classified as negative by the binary classifier of the tool. RODEO was not run on the precursors of the other strains.

Table S6. Biosynthetic Novelty Index (BiNI) of the twelve *Jiangella genomes* included in the comparative analysis. BGC regions were predicted with antiSMASH v8.0.4 under identical settings and submitted to BiG-FAM; the index is the sum of the distances exceeding the novelty threshold (d > 900) divided by the total number of regions. "Fragmented" indicates regions reported by BiG-FAM as truncated by a contig boundary. Genomes are ordered by decreasing BiNI.

Genome	BGC regions (n)	Regions with d > 900	Fragmented regions	BiNI
Jiangella mangrovi DSM 102122	11	11	0	1217.4
Jiangella gansuensis DSM 44835	9	7	0	904.4
Jiangella endophytica KE2-3	11	8	0	884.9
***Jiangella* sp. GA3**	**12**	**9**	**0**	**865.3**
Jiangella asiatica 5K138	10	8	5	855.3
Jiangella muralis DSM 45357	11	8	1	805.5
Jiangella ureilytica KC603	7	5	5	769.7
Jiangella sp. DSM 45060	11	7	0	754.5
Jiangella aurantiaca 8K307	10	6	7	638.1
Jiangella alba DSM 45237	13	7	0	625.2
Jiangella alkaliphila DSM 45079	9	5	0	612.8
Jiangella anatolica GTF31	10	3	6	335.0

Table S7. Assignment of the 124 antiSMASH regions of the twelve *Jiangella genomes* to gene cluster families (GCFs) delineated with BiG-SCAPE v2.0.3 at distance cut-offs of 0.3 and 0.5. For each region the antiSMASH product class, the family identifier, the number of genomes carrying that family and, for the 0.3 cut-off, the resulting category are given. Family FAM_00061, referred to in the text as the mycofactocin family, comprises six regions classified by antiSMASH as redox-cofactor and one, in *Jiangella anatolica*, classified as a ranthipeptide.

Genome	Region	antiSMASH class	GCF (c 0.3)	Genomes (c 0.3)	Category (c 0.3)	GCF (c 0.5)	Genomes (c 0.5)
Jiangella alba	NZ_FNUC01000003.1.region001	lanthipeptide-class-v	FAM_00054	3	rare	FAM_00010	3
Jiangella alba	NZ_FNUC01000003.1.region002	hydrogen-cyanide	FAM_00059	10	common	FAM_00013	10
Jiangella alba	NZ_FNUC01000003.1.region003	RRE-containing.azole-containing-RiPP	FAM_00088	1	strain-specific	FAM_00031	1
Jiangella alba	NZ_FNUC01000003.1.region004	NAGGN	FAM_00112	1	strain-specific	FAM_00003	6
Jiangella alba	NZ_FNUC01000003.1.region005	RiPP-like	FAM_00053	3	rare	FAM_00008	7
Jiangella alba	NZ_FNUC01000003.1.region006	terpene-precursor	FAM_00058	7	common	FAM_00012	12
Jiangella alba	NZ_FNUC01000003.1.region007	T3PKS	FAM_00070	1	strain-specific	FAM_00020	1
Jiangella alba	NZ_FNUC01000004.1.region001	NI-siderophore	FAM_00115	1	strain-specific	FAM_00006	3
Jiangella alba	NZ_FNUC01000004.1.region002	lassopeptide	FAM_00092	1	strain-specific	FAM_00033	1
Jiangella alba	NZ_FNUC01000004.1.region003	redox-cofactor	FAM_00061	7	common	FAM_00015	7
Jiangella alba	NZ_FNUC01000004.1.region004	lassopeptide	FAM_00051	4	common	FAM_00004	6
Jiangella alba	NZ_FNUC01000004.1.region005	RRE-containing	FAM_00097	1	strain-specific	FAM_00007	6
Jiangella alba	NZ_FNUC01000004.1.region006	NRPS-like	FAM_00048	6	common	FAM_00001	9

Jiangella alkaliphila	NZ_LT629791.1.region001	NI-siderophore	FAM_00091	1	strain-specific	FAM_00005	8
Jiangella alkaliphila	NZ_LT629791.1.region002	lanthipeptide-class-i	FAM_00099	1	strain-specific	FAM_00036	1
Jiangella alkaliphila	NZ_LT629791.1.region003	lassopeptide	FAM_00102	1	strain-specific	FAM_00039	1
Jiangella alkaliphila	NZ_LT629791.1.region004	redox-cofactor	FAM_00061	7	common	FAM_00015	7
Jiangella alkaliphila	NZ_LT629791.1.region005	NAGGN	FAM_00063	4	common	FAM_00003	6
Jiangella alkaliphila	NZ_LT629791.1.region006	RiPP-like	FAM_00082	1	strain-specific	FAM_00009	3
Jiangella alkaliphila	NZ_LT629791.1.region007	terpene-precursor	FAM_00057	5	common	FAM_00012	12
Jiangella alkaliphila	NZ_LT629791.1.region008	T3PKS	FAM_00108	1	strain-specific	FAM_00042	1
Jiangella alkaliphila	NZ_LT629791.1.region009	hydrogen-cyanide	FAM_00059	10	common	FAM_00013	10
Jiangella anatolica	NZ_POTW01000001.1.region001	T3PKS	FAM_00085	1	strain-specific	FAM_00013	10
Jiangella anatolica	NZ_POTW01000005.1.region001	RiPP-like	FAM_00104	1	strain-specific	FAM_00009	3
Jiangella anatolica	NZ_POTW01000008.1.region001	lassopeptide	FAM_00075	1	strain-specific	FAM_00011	2
Jiangella anatolica	NZ_POTW01000034.1.region001	NI-siderophore	FAM_00094	1	strain-specific	FAM_00005	8
Jiangella anatolica	NZ_POTW01000063.1.region001	hydrogen-cyanide	FAM_00059	10	common	FAM_00013	10
Jiangella anatolica	NZ_POTW01000069.1.region001	RRE-containing	FAM_00071	1	strain-specific	FAM_00021	1
Jiangella anatolica	NZ_POTW01000102.1.region001	NRPS-like	FAM_00049	3	rare	FAM_00001	9
Jiangella anatolica	NZ_POTW01000108.1.region001	NAGGN	FAM_00050	5	common	FAM_00002	5
Jiangella anatolica	NZ_POTW01000123.1.region001	terpene-precursor	FAM_00057	5	common	FAM_00012	12
Jiangella anatolica	NZ_POTW01000133.1.region001	ranthipeptide	FAM_00061	7	common	FAM_00015	7
Jiangella asiatica	NZ_SMKZ01000003.1.region001	RiPP-like	FAM_00116	1	strain-specific	FAM_00047	1
Jiangella asiatica	NZ_SMKZ01000003.1.region002	T3PKS	FAM_00095	1	strain-specific	FAM_00035	1
Jiangella asiatica	NZ_SMKZ01000006.1.region001	NI-siderophore	FAM_00086	1	strain-specific	FAM_00005	8
Jiangella asiatica	NZ_SMKZ01000013.1.region001	lanthipeptide-class-ii	FAM_00080	1	strain-specific	FAM_00026	1
Jiangella asiatica	NZ_SMKZ01000020.1.region001	lanthipeptide-class-iii	FAM_00062	2	rare	FAM_00016	3
Jiangella asiatica	NZ_SMKZ01000030.1.region001	lassopeptide	FAM_00098	1	strain-specific	FAM_00004	6
Jiangella asiatica	NZ_SMKZ01000063.1.region001	RRE-containing	FAM_00052	2	rare	FAM_00007	6
Jiangella asiatica	NZ_SMKZ01000064.1.region001	hydrogen-cyanide	FAM_00106	1	strain-specific	FAM_00040	1
Jiangella asiatica	NZ_SMKZ01000078.1.region001	NAGGN	FAM_00050	5	common	FAM_00002	5
Jiangella asiatica	NZ_SMKZ01000082.1.region001	terpene-precursor	FAM_00058	7	common	FAM_00012	12
Jiangella aurantiaca	NZ_SMLB01000001.1.region001	hydrogen-cyanide	FAM_00059	10	common	FAM_00013	10
Jiangella aurantiaca	NZ_SMLB01000006.1.region001	terpene-precursor	FAM_00058	7	common	FAM_00012	12
Jiangella aurantiaca	NZ_SMLB01000007.1.region001	lanthipeptide-class-iii	FAM_00062	2	rare	FAM_00016	3
Jiangella aurantiaca	NZ_SMLB01000011.1.region001	NRPS-like	FAM_00048	6	common	FAM_00001	9
Jiangella aurantiaca	NZ_SMLB01000017.1.region001	RiPP-like	FAM_00073	1	strain-specific	FAM_00008	7
Jiangella aurantiaca	NZ_SMLB01000019.1.region001	NI-siderophore	FAM_00064	2	rare	FAM_00005	8
Jiangella aurantiaca	NZ_SMLB01000036.1.region001	NAGGN	FAM_00050	5	common	FAM_00002	5
Jiangella aurantiaca	NZ_SMLB01000080.1.region001	RiPP-like	FAM_00113	1	strain-specific	FAM_00045	1
Jiangella aurantiaca	NZ_SMLB01000081.1.region001	T3PKS	FAM_00100	1	strain-specific	FAM_00037	1
Jiangella aurantiaca	NZ_SMLB01000083.1.region001	T3PKS	FAM_00090	1	strain-specific	FAM_00032	1
Jiangella endophytica	NZ_QVAI01000001.1.region001	lanthipeptide-class-v	FAM_00054	3	rare	FAM_00010	3

Jiangella endophytica	NZ_QVAI01000001.1.region002	hydrogen-cyanide	FAM_00059	10	common	FAM_00013	10
Jiangella endophytica	NZ_QVAI01000005.1.region001	NI-siderophore	FAM_00067	1	strain-specific	FAM_00005	8
Jiangella endophytica	NZ_QVAI01000006.1.region001	redox-cofactor	FAM_00061	7	common	FAM_00015	7
Jiangella endophytica	NZ_QVAI01000008.1.region001	T3PKS	FAM_00069	1	strain-specific	FAM_00019	1
Jiangella endophytica	NZ_QVAI01000009.1.region001	NAGGN	FAM_00096	1	strain-specific	FAM_00003	6
Jiangella endophytica	NZ_QVAI01000010.1.region001	terpene-precursor	FAM_00057	5	common	FAM_00012	12
Jiangella endophytica	NZ_QVAI01000011.1.region001	RRE-containing.azole-containing-RiPP	FAM_00084	1	strain-specific	FAM_00029	1
Jiangella endophytica	NZ_QVAI01000011.1.region002	RiPP-like	FAM_00074	1	strain-specific	FAM_00008	7
Jiangella endophytica	NZ_QVAI01000013.1.region001	RiPP-like	FAM_00068	1	strain-specific	FAM_00018	1
Jiangella endophytica	NZ_QVAI01000017.1.region001	NRPS-like	FAM_00048	6	common	FAM_00001	9
Jiangella gansuensis DSM 44835	NZ_KI911782.1.region001	NI-siderophore	FAM_00093	1	strain-specific	FAM_00034	1
Jiangella gansuensis DSM 44835	NZ_KI911782.1.region002	lassopeptide	FAM_00079	1	strain-specific	FAM_00004	6
Jiangella gansuensis DSM 44835	NZ_KI911782.1.region003	RRE-containing	FAM_00052	2	rare	FAM_00007	6
Jiangella gansuensis DSM 44835	NZ_KI911782.1.region004	lanthipeptide-class-iii	FAM_00117	1	strain-specific	FAM_00016	3
Jiangella gansuensis DSM 44835	NZ_KI911782.1.region005	T3PKS	FAM_00083	1	strain-specific	FAM_00028	1
Jiangella gansuensis DSM 44835	NZ_KI911782.1.region006	terpene-precursor	FAM_00058	7	common	FAM_00012	12
Jiangella gansuensis DSM 44835	NZ_KI911782.1.region007	lanthipeptide-class-iv	FAM_00078	1	strain-specific	FAM_00025	1
Jiangella gansuensis DSM 44835	NZ_KI911782.1.region008	hydrogen-cyanide	FAM_00059	10	common	FAM_00013	10
Jiangella gansuensis DSM 44835	NZ_KI911782.1.region009	NAGGN	FAM_00050	5	common	FAM_00002	5
Jiangella mangrovi	NZ_JACHMM010000001.1.region001	RRE-containing	FAM_00077	1	strain-specific	FAM_00024	1
Jiangella mangrovi	NZ_JACHMM010000001.1.region002	lassopeptide	FAM_00111	1	strain-specific	FAM_00011	2
Jiangella mangrovi	NZ_JACHMM010000001.1.region003	NI-siderophore	FAM_00089	1	strain-specific	FAM_00006	3
Jiangella mangrovi	NZ_JACHMM010000001.1.region004	oligosaccharide	FAM_00101	1	strain-specific	FAM_00038	1
Jiangella mangrovi	NZ_JACHMM010000001.1.region005	hydrogen-cyanide	FAM_00059	10	common	FAM_00013	10
Jiangella mangrovi	NZ_JACHMM010000001.1.region006	T3PKS	FAM_00076	1	strain-specific	FAM_00023	1
Jiangella mangrovi	NZ_JACHMM010000001.1.region007	terpene-precursor	FAM_00057	5	common	FAM_00012	12
Jiangella mangrovi	NZ_JACHMM010000001.1.region008	lassopeptide	FAM_00072	1	strain-specific	FAM_00022	1
Jiangella mangrovi	NZ_JACHMM010000001.1.region009	RiPP-like	FAM_00056	2	rare	FAM_00008	7
Jiangella mangrovi	NZ_JACHMM010000001.1.region010	NAGGN	FAM_00063	4	common	FAM_00003	6
Jiangella mangrovi	NZ_JACHMM010000001.1.region011	NRPS-like	FAM_00049	3	rare	FAM_00001	9
Jiangella muralis	NZ_KQ434751.1.region001	terpene-precursor	FAM_00058	7	common	FAM_00012	12
Jiangella muralis	NZ_KQ434755.1.region001	T3PKS	FAM_00081	1	strain-specific	FAM_00027	1
Jiangella muralis	NZ_KQ434756.1.region001	NI-siderophore	FAM_00064	2	rare	FAM_00005	8
Jiangella muralis	NZ_KQ434757.1.region001	redox-cofactor	FAM_00061	7	common	FAM_00015	7
Jiangella muralis	NZ_LFXL01000010.1.region001	NRPS-like	FAM_00048	6	common	FAM_00001	9

Jiangella muralis	NZ_LFXL01000025.1.region001	NAGGN	FAM_00050	5	common	FAM_00002	5
Jiangella muralis	NZ_LFXL01000030.1.region001	RiPP-like	FAM_00053	3	rare	FAM_00008	7
Jiangella muralis	NZ_LFXL01000036.1.region001	lanthipeptide-class-i	FAM_00107	1	strain-specific	FAM_00041	1
Jiangella muralis	NZ_LFXL01000039.1.region001	lassopeptide	FAM_00051	4	common	FAM_00004	6
Jiangella muralis	NZ_LFXL01000045.1.region001	hydrogen-cyanide	FAM_00059	10	common	FAM_00013	10
Jiangella muralis	NZ_LFXL01000054.1.region001	RRE-containing	FAM_00055	3	rare	FAM_00007	6
Jiangella sp. GA3	GA3_1.region001	NI-siderophore	FAM_00065	2	rare	FAM_00005	8
Jiangella sp. GA3	GA3_1.region002	hydrogen-cyanide	FAM_00059	10	common	FAM_00013	10
Jiangella sp. GA3	GA3_14.region001	RRE-containing	FAM_00055	3	rare	FAM_00007	6
Jiangella sp. GA3	GA3_2.region001	lassopeptide	FAM_00051	4	common	FAM_00004	6
Jiangella sp. GA3	GA3_2.region002	redox-cofactor	FAM_00061	7	common	FAM_00015	7
Jiangella sp. GA3	GA3_5.region001	terpene-precursor	FAM_00058	7	common	FAM_00012	12
Jiangella sp. GA3	GA3_5.region002	T3PKS	FAM_00109	1	strain-specific	FAM_00043	1
Jiangella sp. GA3	GA3_6.region001	NAGGN	FAM_00063	4	common	FAM_00003	6
Jiangella sp. GA3	GA3_6.region002	other	FAM_00066	1	strain-specific	FAM_00017	1
Jiangella sp. GA3	GA3_6.region003	NRPS-like	FAM_00048	6	common	FAM_00001	9
Jiangella sp. GA3	GA3_7.region001	RiPP-like	FAM_00060	2	rare	FAM_00014	2
Jiangella sp. GA3	GA3_8.region001	RiPP-like	FAM_00053	3	rare	FAM_00008	7
Jiangella sp. DSM 45060	NZ_LT629771.1.region001	lanthipeptide-class-v	FAM_00054	3	rare	FAM_00010	3
Jiangella sp. DSM 45060	NZ_LT629771.1.region002	hydrogen-cyanide	FAM_00059	10	common	FAM_00013	10
Jiangella sp. DSM 45060	NZ_LT629771.1.region003	terpene-precursor	FAM_00058	7	common	FAM_00012	12
Jiangella sp. DSM 45060	NZ_LT629771.1.region004	T3PKS	FAM_00110	1	strain-specific	FAM_00044	1
Jiangella sp. DSM 45060	NZ_LT629771.1.region005	RiPP-like	FAM_00103	1	strain-specific	FAM_00009	3
Jiangella sp. DSM 45060	NZ_LT629771.1.region006	RiPP-like	FAM_00060	2	rare	FAM_00014	2
Jiangella sp. DSM 45060	NZ_LT629771.1.region007	NRPS-like	FAM_00048	6	common	FAM_00001	9
Jiangella sp. DSM 45060	NZ_LT629771.1.region008	RRE-containing	FAM_00055	3	rare	FAM_00007	6
Jiangella sp. DSM 45060	NZ_LT629771.1.region009	lassopeptide	FAM_00051	4	common	FAM_00004	6
Jiangella sp. DSM 45060	NZ_LT629771.1.region010	redox-cofactor	FAM_00061	7	common	FAM_00015	7
Jiangella sp. DSM 45060	NZ_LT629771.1.region011	NI-siderophore	FAM_00065	2	rare	FAM_00005	8
Jiangella ureilytica	NZ_SMKL01000004.1.region001	oligosaccharide	FAM_00114	1	strain-specific	FAM_00046	1
Jiangella ureilytica	NZ_SMKL01000008.1.region001	NI-siderophore	FAM_00105	1	strain-specific	FAM_00006	3
Jiangella ureilytica	NZ_SMKL01000009.1.region001	NRPS-like	FAM_00049	3	rare	FAM_00001	9
Jiangella ureilytica	NZ_SMKL01000012.1.region001	T3PKS	FAM_00087	1	strain-specific	FAM_00030	1
Jiangella ureilytica	NZ_SMKL01000028.1.region001	RiPP-like	FAM_00056	2	rare	FAM_00008	7
Jiangella ureilytica	NZ_SMKL01000067.1.region001	terpene-precursor	FAM_00057	5	common	FAM_00012	12
Jiangella ureilytica	NZ_SMKL01000092.1.region001	NAGGN	FAM_00063	4	common	FAM_00003	6

Table S8. Genome assemblies of the genus *Jiangella retrieved* from NCBI RefSeq and used in the comparative analysis of biosynthetic gene clusters. Assemblies with more than 300 contigs

were excluded, and where the same strain was represented by more than one assembly the more contiguous one was retained.

Species	Strain	Type strain	Assembly accession	Contigs	Genome size (bp)	N50 (bp)	Status
Jiangella sp. GA3	GA3		this study	27	7,462,732	886,138	included (this study)
Jiangella alba	YIM 61503	T	GCF_001708125.1	33	7,664,864	390,506	excluded — redundant assembly of the same strain (less contiguous)
Jiangella alba	DSM 45237 (= YIM 61503)	T	GCF_900106035.1	4	7,681,885	3,325,316	included
Jiangella alkaliphila	KCTC 19222	T	GCF_001005145.1	124	7,567,836	169,179	excluded — redundant assembly of the same strain (less contiguous)
Jiangella alkaliphila	DSM 45079	T	GCF_900105925.1	1	7,716,600	7,716,600	included
Jiangella anatolica	GTF31	T	GCF_003236295.1	256	7,005,973	54,053	included
Jiangella asiatica	5K138	T	GCF_004349065.1	134	7,034,063	116,826	included
Jiangella aurantiaca	8K307	T	GCF_004349105.1	102	6,620,775	118,582	included
Jiangella endophytica	KE2-3	T	GCF_003427025.1	89	7,850,704	298,984	included
Jiangella gansuensis	DSM 44835	T	GCF_000515395.1	1	5,585,780	5,585,780	included
Jiangella mangrovi	DSM 102122	T	GCF_014204975.1	1	7,164,547	7,164,547	included
Jiangella muralis	DSM 45357	T	GCF_001270745.1	76	7,266,157	324,980	included
Jiangella rhizosphaerae	NEAU-YY265	T	GCF_003579925.1	441	6,500,878	28,547	excluded — >300 contigs (assembly quality)
Jiangella sp. DSM 45060	DSM 45060		GCF_900105175.1	1	7,486,057	7,486,057	included
Jiangella ureilytica	KC603	T	GCF_004348545.1	168	6,345,964	76,365	included
